# Improved simultaneous mapping of epigenetic features and 3D chromatin structure via ViCAR

**DOI:** 10.1186/s13059-024-03377-6

**Published:** 2024-09-03

**Authors:** Sean M. Flynn, Somdutta Dhir, Krzysztof Herka, Colm Doyle, Larry Melidis, Angela Simeone, Winnie W. I. Hui, Rafael de Cesaris Araujo Tavares, Stefan Schoenfelder, David Tannahill, Shankar Balasubramanian

**Affiliations:** 1 Cancer Research UK Cambridge Institute, Li Ka Shing Centre, Robinson Way, Cambridge, CB2 0RE UK; 2 Epigenetics Programme, Babraham Institute, Cambridge, CB22 3AT UK; 3 Yusuf Hamied Department of Chemistry, University of Cambridge, Cambridge, CB2 1EW UK; 4 School of Clinical Medicine, University of Cambridge, Cambridge, CB2 0SP UK

**Keywords:** 3D genome structure, Hi-C, Histone marks, G-quadruplex DNA

## Abstract

Methods to measure chromatin contacts at genomic regions bound by histone modifications or proteins are important tools to investigate chromatin organization. However, such methods do not capture the possible involvement of other epigenomic features such as G-quadruplex DNA secondary structures (G4s). To bridge this gap, we introduce ViCAR (viewpoint HiCAR), for the direct antibody-based capture of chromatin interactions at folded G4s. Through ViCAR, we showcase the first G4-3D interaction landscape. Using histone marks, we also demonstrate how ViCAR improves on earlier approaches yielding increased signal-to-noise. ViCAR is a practical and powerful tool to explore epigenetic marks and 3D genome interactomes.

**Supplementary Information:**

The online version contains supplementary material available at 10.1186/s13059-024-03377-6.

## Background

Chromosome conformation capture methodologies, such as Hi-C [1], provide information on 3D genome structure and function including the role of DNA looping in facilitating enhancer function [2]. Genome-wide Hi-C maps require prohibitively high sequencing depth (often requiring billions of reads), so more pragmatic methods such as Capture Hi-C [3, 4], Hi-C on accessible regulatory DNA (HiCAR) [5], Hi-C Coupled chromatin cleavage and Tagmentation (HiCuT) [6], Proximity Ligation-Assisted ChIP-seq (PLAC-seq) [7], and Hi-C with Chromatin Immunoprecipitation (HiChIP) [8] have been developed to map 3D interactions for specific genome features. For example, HiCAR uses Tn5 transposase activity to tag 3D interactions anchored in accessible genome regions [5].

G-quadruplexes (G4s) are four-stranded structures that can fold in specific G-rich DNA sequences [9, 10]. Folded G4s have been detected in thousands of gene regulatory regions in human chromatin [11]. Chromatin immunoprecipitation followed by sequencing (ChIP-seq) [11] and Cleavage Under Targets and Tagmentation (CUT&Tag) [12–16] using G4-specific antibodies or small molecules have generated maps of folded G4 sites in different cell types, states, and diseases. G4s are enriched in active promoters and enhancers, and overlapping these data with independently generated 3D interaction maps suggests that G4s may associate with sites of promoter-enhancer contact [17–20]. Emerging evidence shows that G4 profiles are remodeled to reflect cell identity and transitions between pluripotent and differentiated states, and normal to cancer states [17, 21]. Taken together, these findings suggest that G4s may have a role to play in promoter-enhancer 3D interactions. However, the experimental evidence for the formation of G4s at enhancer-promoter contact sites is not direct and is based on indirect correlations by overlapping independent datasets. A methodology that can detect folded G4 directly at promoter-enhancer interaction sites, in the same DNA fragment (i.e., at read level), would reveal the co-occurrence of G4s at enhancers more convincingly. Herein, we introduce such a method.

## Results and discussion

**Fig. 1:**
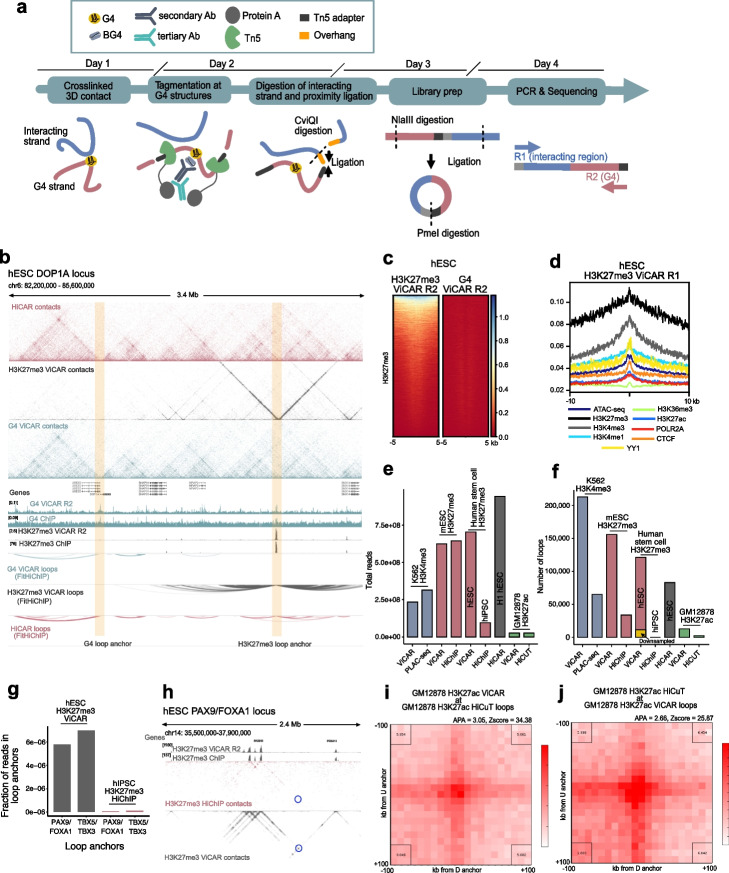
**a** Overview of the ViCAR method. Tn5 tagments at sites where an antibody binds the desired feature, in this example a folded G4 structure. Subsequently, the genome is digested with CviQI and tagmented and digested fragments in spatial proximity ligated. To amplify ligated fragments, an i7 primer that anneals to the Tn5 mosaic end adapter and an i5 primer that anneals to a splint oligonucleotide ligated to genomic DNA are used for PCR. The i7 ends of PCR products are sequenced as R2, and the i5 ends are sequenced as R1. **b** Example locus from ViCAR and HiCAR [5] data for H1 hESCs. Top 3 tracks show raw contact matrices; 2D tracks show ViCAR R2 and ChIP-seq for H3K27me3 and G4s in hESCs. Bottom 3 tracks show significant loops called by FitHiChIP (*q* < 0.05). Highlighted regions are examples of G4- and H3K27me3-centered loop anchors. **c** H3K27me3 ViCAR sequencing R2 plotted over H3K27me3 ChIP-seq peaks (ENCODE) in hESCs (left). G4 ViCAR sequencing R2 over H3K27me3 ChIP-seq peaks is shown for comparison (right). **d** H3K27me3 ViCAR sequencing R1 (i.e., 3D interactions) plotted over ChIP-seq and ATAC-seq peaks. Sequencing depth (**e**) and number of significant loops called by FitHiChIP (**f**) in ViCAR and other methods. The yellow bar in **f** represents hESC H3K27me3 ViCAR data down-sampled to the same number of valid read pairs as the hiPSC H3K27me3 HiChIP data (11,414,395 pairs). **g** and** h** Comparison of hESC H3K27me3 ViCAR and hiPSC H3K27me3 HiChIP data [23] at the PAX9/FOXA1 (chr14: 36,660,000–36,670,000/chr14: 37,590,000–37,600,000) and TBX5/TBX3 (chr12: 114,400,000–114,410,000/chr12: 114,680,000–114,690,000) loci highlighted by Kraft et al. [23]. **g** shows number of reads underlying PAX9/FOXA1 and TBX5/TBX3 loops as a fraction of total number of reads per library. Loops were called by FitHiChIP at 10 kb resolution. **h** shows raw contact matrices for the PAX9/FOXA1 locus. **i** APA plot for GM12878 H3K27ac HiCuT [6] loops using GM12878 H3K27ac ViCAR data. **j** APA plot for GM12878 H3K27ac ViCAR loops using GM12878 H3K27ac HiCuT data [6]

We have developed ViCAR (viewpoint HiCAR) to overcome two major limitations of existing methodologies that are not amenable to mapping G4s directly at 3D contact sites. We aimed to improve (1) the signal-to-noise seen in ChIP-based technologies by deploying CUT&Tag [6, 14, 22] and (2) HiCuT, by enriching ligated fragments, which HiCuT does not do. HiCuT is the only CUT&Tag-based Hi-C method that we know of, but it only has a low proportion of useful reads (cis interactions > 20 kb; Additional file 2: Table S1) [6]. G4 ViCAR works by recruiting Tn5 to folded G4 structures present in chromatin using a G4-specific antibody. Tagmentation is then activated, and subsequent restriction digestion with CviQI followed by ligation connects DNA fragments proximal to the tagmented G4 site. Enrichment of ligation junctions is performed via PCR using one primer that anneals to the mosaic end region of the Tn5 adapter, and another that anneals to a splint oligonucleotide which is ligated to genomic DNA [5] (Fig. 1a). The G4 anchored loop site in the tagmented DNA fragment is then sequenced as Read 2 (R2), while the region that was in spatial proximity is sequenced as Read 1 (Fig. 1a).

To demonstrate the improved efficiency of ViCAR, we first used antibodies for histone marks to provide a comparison with maps of 3D genome structure by HiChIP, PLAC-seq, or HiCuT [6, 23, 24]. We performed ViCAR for the H3K27me3 repressive mark in human embryonic stem cells (hESCs) and mouse embryonic stem cells (mESCs), which captures loops at developmental genes with low levels of expression [25]. Tagmentation of antibody-bound sites was confirmed by the ViCAR R2 signal, which reproducibly enriched H3K27me3 sites as compared to previous hESC (ENCODE) and mESC [26] ChIP-seq data [17] (Fig. 1b–c; Additional file 1: Fig. S1a–d). As expected, ViCAR validated homotypic 3D interactions marked by H3K27me3 on both sides in hESCs [27] (Fig. 1d). Using FitHiChIP [28] to call loops, we identified 161,819 H3K27me3-anchored loops at 5 kb resolution in hESCs (Additional file 1: Fig. S1e–f, Additional file 2: Table S1). Known H3K27me3 loops anchored at HOX clusters and known inter-chromosomal interactions were clearly exemplified by ViCAR (Additional file 1: Fig. S2a–c) [27]. The improved efficiency of ViCAR was clear from side-by-side comparison of H3K27me3 ViCAR to H3K27me3 HiChIP data for mESCs and human induced pluripotent stem cells (hiPSCs) [23]. In mESCs, ViCAR identified 284,174 significant loops, whereas published HiChIP [23] only identified 34,597 at a similar sequencing depth. As published HiChIP data from hiPSCs was sequenced to lower depth than ViCAR, for a fair comparison ViCAR data from hESCs was down-sampled to the same number of valid pairs as HiChIP before loop calling. Notably, ViCAR identified 11,433 significant loops, whereas no significant loops were detectable with HiChIP (Fig. 1e–f). On analyzing raw interactions, we found that ViCAR consistently identifies contacts with strikingly improved signal-to-noise compared to HiChIP at the PAX9/FOXA1 and TBX5/TBX3 loci (Fig. 1g–h; Additional file 1: Fig. S2d). Overall, these results demonstrate the improved sensitivity of ViCAR relative to HiChIP for identifying 3D genome interactions.

To further show the advantage of ViCAR over PLAC-seq, we performed VICAR for the H3K4me3 promoter mark and H3K4me1 enhancer mark in human K562 erythroleukemia cells which have extensive 3D genome maps [24, 29]. We validated ViCAR at the well-characterized MYC locus [24, 29–31], and confirmed that the ViCAR 3D structure was consistent with published in situ Hi-C [29] and PLAC-seq [24] data, and that H3K4me3 ViCAR recovered known MYC promoter-enhancer loops [31] (Additional file 1: Fig. S3a–b). At comparable sequencing depth, ViCAR detects more H3K4me3-anchored loops than PLAC-seq [24] (212,520 vs 65,005; Fig. 1g–h; Additional file 1: Fig. S3c–d; Additional file 2: Table S1). Together, these data show that ViCAR provides increased sensitivity for loop detection compared to HiChIP and PLAC-seq.

To compare ViCAR to HiCuT directly, we performed ViCAR in GM12878 cells using the H3K27ac antibody and down-sampled it to a similar number of reads for published HiCuT H3K27ac data in the same cell type [6] (Additional file 2: Table S1). Whereas in HiCuT data 1.89% of total reads were useful for Hi-C (cis interactions > 20 kb), 30.79% of total reads met the same criteria in ViCAR data (Additional file 2: Table S1). Using FitHiChIP, we identified 12,904 loops with ViCAR, compared to 2639 loops with HiCuT (10 kb resolution, *q* < 0.05; Additional file 2: Table S1). Therefore, ViCAR provides increased sensitivity and represents a significant advance compared to HiCuT.

To further benchmark our method against existing approaches, we confirmed that the size of the loops identified by ViCAR are comparable to those called with HiChIP, PLAC-seq, HiCuT, and HiCAR (Additional file 1: Fig. S4a–h). We also used aggregate peak analysis (APA) [29] to compare enrichment of sites identified by ViCAR and other methods. APA plots showed that HiCuT [6], PLAC-seq [24], and HiChIP [23] loops were enriched in ViCAR data (Fig. 1i and Additional file 1: Fig. S4i–j), suggesting that interactions detected by other methods are also identified by ViCAR. ViCAR loops were also enriched in HiCuT, PLAC-seq, and HiChIP data (Fig. 1j and Additional file 1: Fig. S4k–l) indicating that ViCAR loops are bona fide. Additionally, loops that were unique to H3K4me3 ViCAR were identified in H3K4me3 PLAC-seq upon removal of a *q* value threshold (Additional file 1: Fig. S4m), supporting this conclusion.

**Fig. 2:**
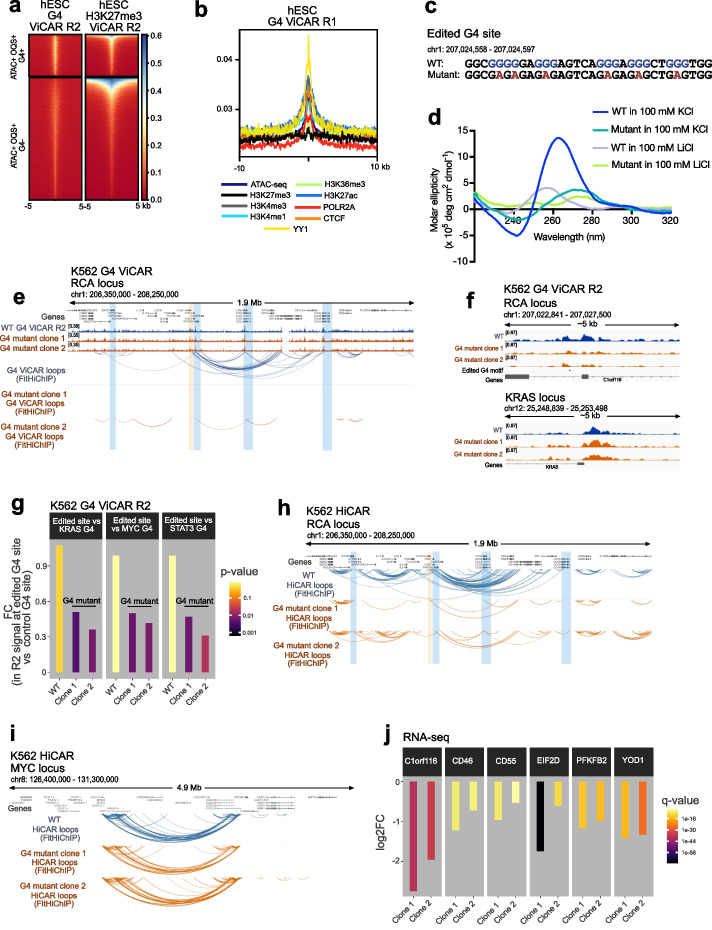
**a** G4 ViCAR sequencing R2 plotted over G4 ChIP-seq peaks [17] in hESCs (left). H3K27me3 ViCAR sequencing R2 plotted over G4 ChIP-seq peaks is shown for comparison (right). Regions with (G4 +) and without (G4 −) G4 ChIP peaks in accessible regions (ATAC-seq peaks; ATAC +) that contain sequences capable of forming G4s in vitro (called Observed Quadruplex Sequences, OQS +) are shown. BG4 ViCAR normalized R2 signal is enriched at G4 + sites compared to G4 − sites. By contrast, H3K27me3 ViCAR normalized R2 signal mostly accumulates at G4 − sites. **b** G4 ViCAR sequencing R1 (i.e., 3D interactions) plotted over ChIP-seq and ATAC-seq peaks. **c** WT and mutant sequence of a G4 oligonucleotide derived from a sequence in an intron of C1orf116. **d** Circular dichroism (CD) spectra of oligonucleotides corresponding to WT and mutant G4 motifs are consistent with a G4 structure in the WT (K^+^-dependent positive peak at ~ 265 nm and negative peak at ~ 240 nm) and a loss of G4 structure in the mutant (with a shift towards 280 nm). **e**–**g** G4 ViCAR data from K562 cells at the edited G4 site in WT and 2 G4 mutant clones. In **e**, the edited G4 site is highlighted by orange shading, and genes affected by the G4 mutation (**j**) are highlighted by blue shading. 2D tracks in **e** show G4 ViCAR R2, and the bottom 3 tracks show loops called by FitHiChIP at 10 kb resolution (*q* < 0.01). **f** G4 ViCAR R2 signal in WT and 2 G4 mutant clones at the edited site. The edited G4 motif is shown by a black bar. **g** Fold change and *p* values represent R2 signal at the edited site vs R2 signal at 3 control G4 sites (KRAS, MYC, STAT3). HiCAR data from K562 cells at the edited G4 site (**h**) and the unedited MYC locus (**i**) in WT and 2 G4 mutant clones. In **h**, the edited G4 site is highlighted by orange shading, and genes affected by the G4 mutation (**j**) are highlighted by blue shading. Loops were called using FitHiChIP at 10 kb resolution (*q* < 0.01). **j** Expression of selected genes near to the edited G4 site measured by RNA-seq

Having established the applicability of ViCAR for histone marks, we next tested the capability of ViCAR to capture additional epigenetic features which have not been directly mapped in 3D genome-wide. To identify loops marked by G4 structures, we performed ViCAR using the G4-specific antibody BG4 in hESCs. Tagmentation near folded G4 sites was confirmed by the ViCAR R2 signal, which was enriched for G4 sites [17] (Figs. 1b and 2a, Additional file 1: Figs. S1b–c and S5a–b). As suggested by previous associations [17, 18, 20], G4 ViCAR also directly confirmed enrichment of CTCF, YY1, and active histone marks at G4-interacting regions (Fig. 2b). Using FitHiChIP [28] to call loops, we identified 9080 G4 loops at 5 kb resolution in hESCs (Additional file 1: Fig. S1e–f, Additional file 2: Table S1). The majority (> 70% with *q* < 0.01) of G4-anchored loops were contained within accessible chromatin HiCAR regions [5] (Additional file 1: Fig. S1e). In contrast, loops anchored by the H3K27me3 repressive mark exhibited little overlap with HiCAR or G4 ViCAR loops (Additional file 1: Fig. S1e–f), demonstrating the depletion of G4s in these regions. Previous comparison of independent ChIP-seq and ChIA-PET (Chromatin Interaction Analysis with Paired-End Tag) data suggests interactions at the KRAS and MDM2 loci [19]. We used ViCAR to directly confirm these G4-3D interactions in K562 cells (Additional file 1: Fig. S5c–d). Overall, our data confirms the existence of G4s at previously predicted sites.

To evaluate whether the G4 ViCAR readout can sense loss of DNA structures in cells, we used CRISPR/Cas9 to remove an endogenous G4 structure in cells. We selected a loop anchor identified above by ViCAR with a short G-rich sequence predicted to form a stable G4. This intronic G4 is located near the regulators of complement activation (RCA) gene cluster. RCA cluster genes including CD55 and CD46 are overexpressed in several malignancies including myeloid leukemia and are a target for cancer therapeutics [32–35], but little is known about how their expression is regulated. Using CRISPR/Cas9, we introduced 6 G > A mutations predicted to abolish G4 structure formation into the endogenous G4 motif in K562 cells (Fig. 2c and Additional file 1: Fig. S6a–c). Biophysical analysis by circular dichroism spectroscopy confirmed that a DNA oligonucleotide of the selected G4 sequence had a signature consistent with G4 formation in 100 mM KCl (positive peak ~ 265 nm, negative peak ~ 240 nm) [36], which is lost in 100 mM LiCl. By contrast the mutated oligonucleotide exhibited a signature consistent with loss of G4 structure (Fig. 2d). Indeed, in edited cells, we observed a reduction in G4 structure (Fig. 2e–g) and G4-associated loops (Fig. 2e) at this locus in two independent clones. Global G4 levels were unchanged (Additional file 1: Fig. S6d), and more loop loss was seen at the RCA locus compared to the remainder of the genome in the mutants (Additional file 1: Fig. S6e–f). Together, this shows that ViCAR will discriminate between unfolded and folded G4s. One limitation of ViCAR, and other immunoprecipitation-based 3D methods, is the dependency on a target feature to probe looping. In cases where the target feature is not available, it is therefore necessary to independently measure changes in the underlying 3D genome. To address what happens to the 3D landscape in the absence of a G4 in the mutant, we performed HiCAR. In edited cells, we observed a reduction in HiCAR loops at the RCA locus, showing that 3D contacts associated with accessible chromatin are reduced upon loss of a G4 (Fig. 2h–i and Additional file 1: Fig. S7a–b). Furthermore, we noted that G4 mutation can perturb gene expression of nearby complement genes CD55 and CD46 as well as C1orf116, YOD1, EIF2D, and PFKFB2 (Fig. 2j and Additional file 3: Table S2), and perturb RNA Pol II occupancy (Additional file 1: Fig. S7c–d). This exemplifies the utility of ViCAR to identify functional distal regulatory regions and pair them with their target genes.

## Conclusions

Here, we introduce ViCAR, which demonstrates several advantages for capturing 3D genome interactions marked by specific epigenetic features. ViCAR shows substantial improvements in sensitivity and significant loop identification compared to other methods. The G4 field to date has relied on indirect correlations to link G4s to 3D genome structure. A major advance is the ability of ViCAR to simultaneously map DNA secondary structures and 3D loops at read-level genome-wide, thus directly demonstrating that they co-occur in the same cell at the same time. ViCAR confirms previously predicted loops and robustly captures G4-3D interactions genome-wide. We anticipate that ViCAR will provide a simple and superior tool to analyze a wide range of factors in 3D genome structure regulation.

## Methods

Methods are provided in Additional file 6.

### Supplementary Information

Additional file 1. Supplementary figures.

Additional file 2: Table S1. ViCAR library statistics.

Additional file 3: Table S2. Differential gene expression analysis (RNA-seq).

Additional file 4: Table S3. Oligonucleotide sequences.

Additional file 5: Table S4. Number of replicates for sequencing experiments.

Additional file 6. Methods [57–64].

Additional file 7. Review history.

## Data Availability

Data generated in this study are deposited in NCBI Gene Expression Omnibus (GEO; https://www.ncbi.nlm.nih.gov/geo/) with accession code GSE250219 [37]. The following data from the 4DN data portal [38] were used: 4DNEXRI3VAH3 (K562 PLAC-seq) [24, 39], 4DNESI7DEJTM (K562 in situ Hi-C) [29, 40]. The following data from Gene Expression Omnibus were used: GSE162819 (hESC HiCAR) [5, 41], GSE150907 (hiPSC H3K27me3 HiChIP and mESC H3K27me3 HiChIP) [23, 42], GSM5658773 (mESC H3K27me3 ChIP-seq) [26, 43], GSE186011 (GM12878 H3K27ac HiCuT) [6, 44], GSE161531 (hESC G4 ChIP-seq and hESC H3K27me3 ChIP-seq) [17, 45], GSE162299 (K562 G4 ChIP-seq) [46, 47]. The following ENCODE datasets were used: ENCFF927FVH (hESC H3K27me3 ChIP-seq) [48, 49], ENCFF368LWM (hESC CTCF ChIP-seq) [50, 51], ENCFF599IHW (hESC H3K36me3 ChIP-seq) [48, 52], ENCFF162HPV (hESC H3K27ac ChIP-seq) [50, 53], ENCFF480QNT (hESC H3K4me1 ChIP-seq) [50, 51], ENCFF120KQK (hESC H3K4me3 ChIP-seq) [50, 51], ENCSR000AKC (GM12878 H3K27ac ChIP-seq) [50, 51], ENCSR000BKD (hESC YY1 ChIP-seq) [53, 54]. Code is available on GitHub [55] and Zenodo [56] under a Creative Commons Zero v1.0 Universal License.
