# Supplementary material for: Improved simultaneous mapping of epigenetic features and 3D chromatin structure via ViCAR
Source: Genome Biol. 2024 Sep 3;25:237. doi: 10.1186/s13059-024-03377-6 (PMC11370281; doi:10.1186/s13059-024-03377-6)
Supplement: Supplementary file 1 — Additional file 1. Supplementary figures. [file 13059_2024_3377_MOESM1_ESM.pdf]

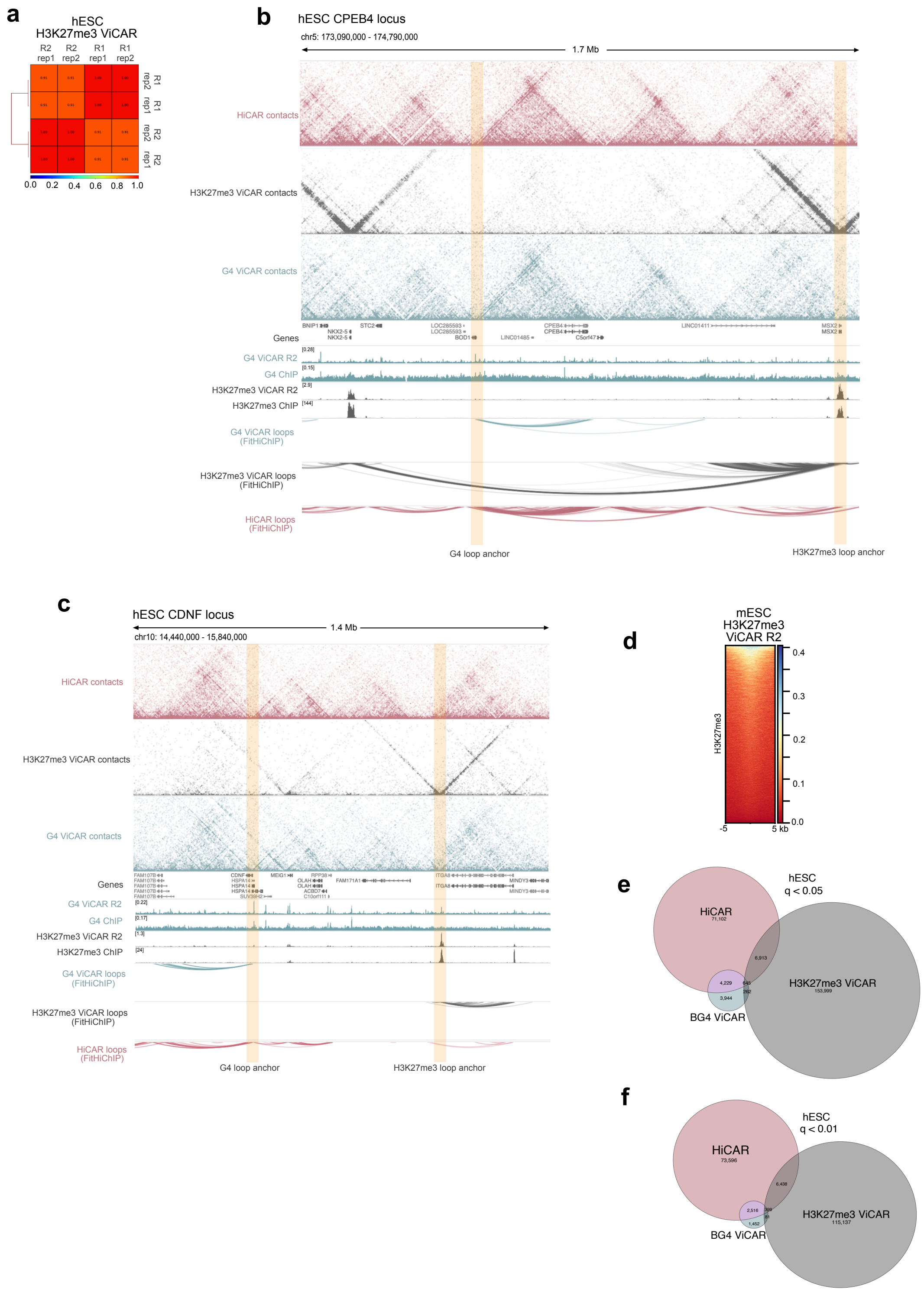

Fig S1. a Reproducibility of ViCAR sequencing reads 1 and 2 (R1 and R2) for H3K27me3 for two replicates (rep1 and rep2) in hESCs. Pearson correlations are shown. **b** and **c** Related to Fig. 1b. Additional examples of ViCAR data from H1 hESCs. Top 3 tracks show raw contact matrices, 2D tracks show ViCAR R2 and ChIP-seq for G4s and H3K27me3 in hESCs, and the bottom 3 tracks show significant loops called by FitHiChIP ($q < 0.05$). Highlighted regions are examples of G4- and H3K27me3- loop anchors. **d** H3K27me3 ViCAR sequencing R2 plotted over

H3K27me3 ChIP-seq peaks [26] in mESCs. **e** and **f** Comparison of loops called by FitHiChIP in HiCAR [5], G4 ViCAR, and H3K27me3 ViCAR in hESCs with significance thresholds of $q < 0.05$ (**e**) and $q < 0.01$ (**f**).

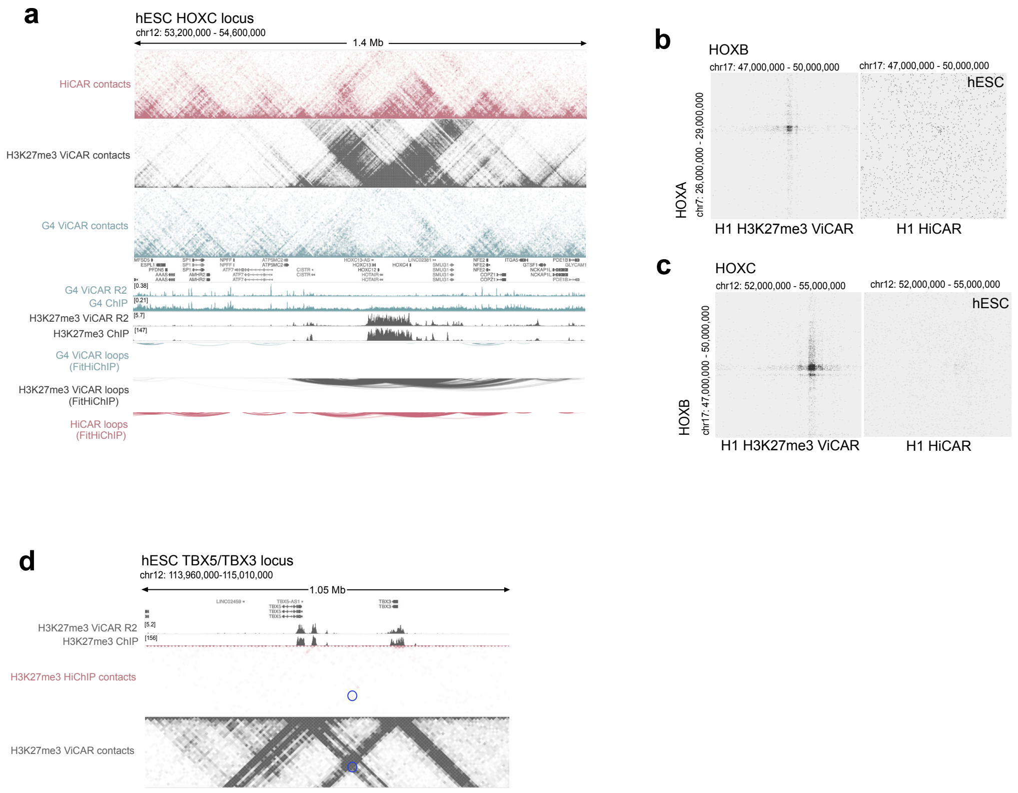

Fig S2. a ViCAR data from H1 hESCs at the HOXC locus**.** Top 3 tracks show raw contact matrices, 2D tracks show ViCAR R2 and ChIP-seq for G4s and H3K27me3 in hESCs, and bottom 3 tracks show significant loops called by FitHiChIP ($q < 0.05$). **b** and **c** Raw contact matrices showing inter-chromosomal (trans) interactions between the HOXA and HOXB clusters (**b**) and HOXB and HOXC clusters (**c**) detected by H3K27me3 ViCAR (left) or HiCAR (right) in H1 hESCs. **d** Related to Fig.1g-h. Comparison of ViCAR and HiChIP data at the TBX5/TBX3 locus highlighted by Kraft et al [23].

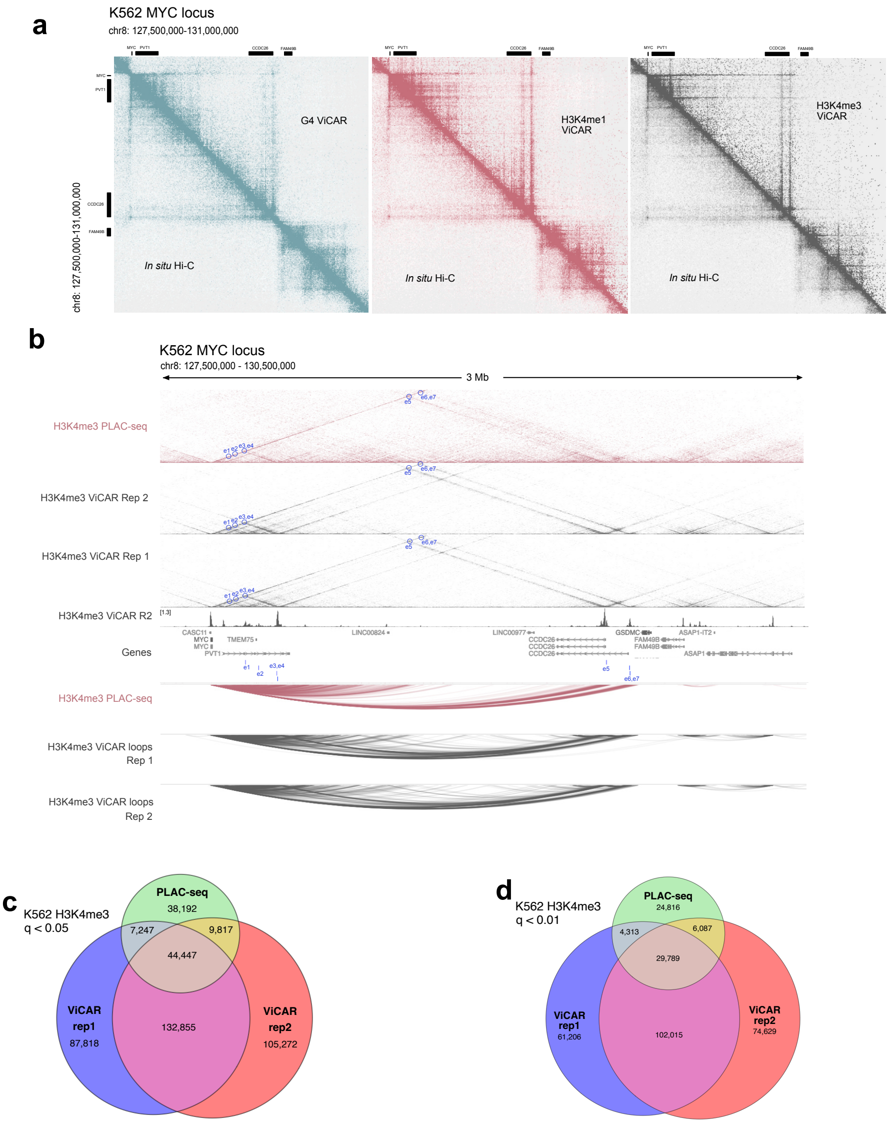

Fig S3. a Raw contact matrices for ViCAR with G4, H3K4me3 and H3K4me1 antibodies showing the MYC locus in K562 cells. **b**, Comparison of PLAC-seq and H3K4me3 in K562 cells. Top 3 tracks show raw contact matrices in PLAC-seq and two replicates of H3K4me3 ViCAR. The 2D track shows ViCAR R2. Bottom 3 tracks show significant loops called by FitHiChIP (q < 0.05) for PLAC-seq and two replicates of H3K4me3 ViCAR. MYC enhancers identified by Fulco et al [31] are shown in blue (e1-e7). **c** and **d** Comparison of loops identified by FitHiChIP in H3K4me3 PLAC-seq (green) and two replicates of H3K4me3 ViCAR in K562 cells. Data are shown at 2 different q-value thresholds.

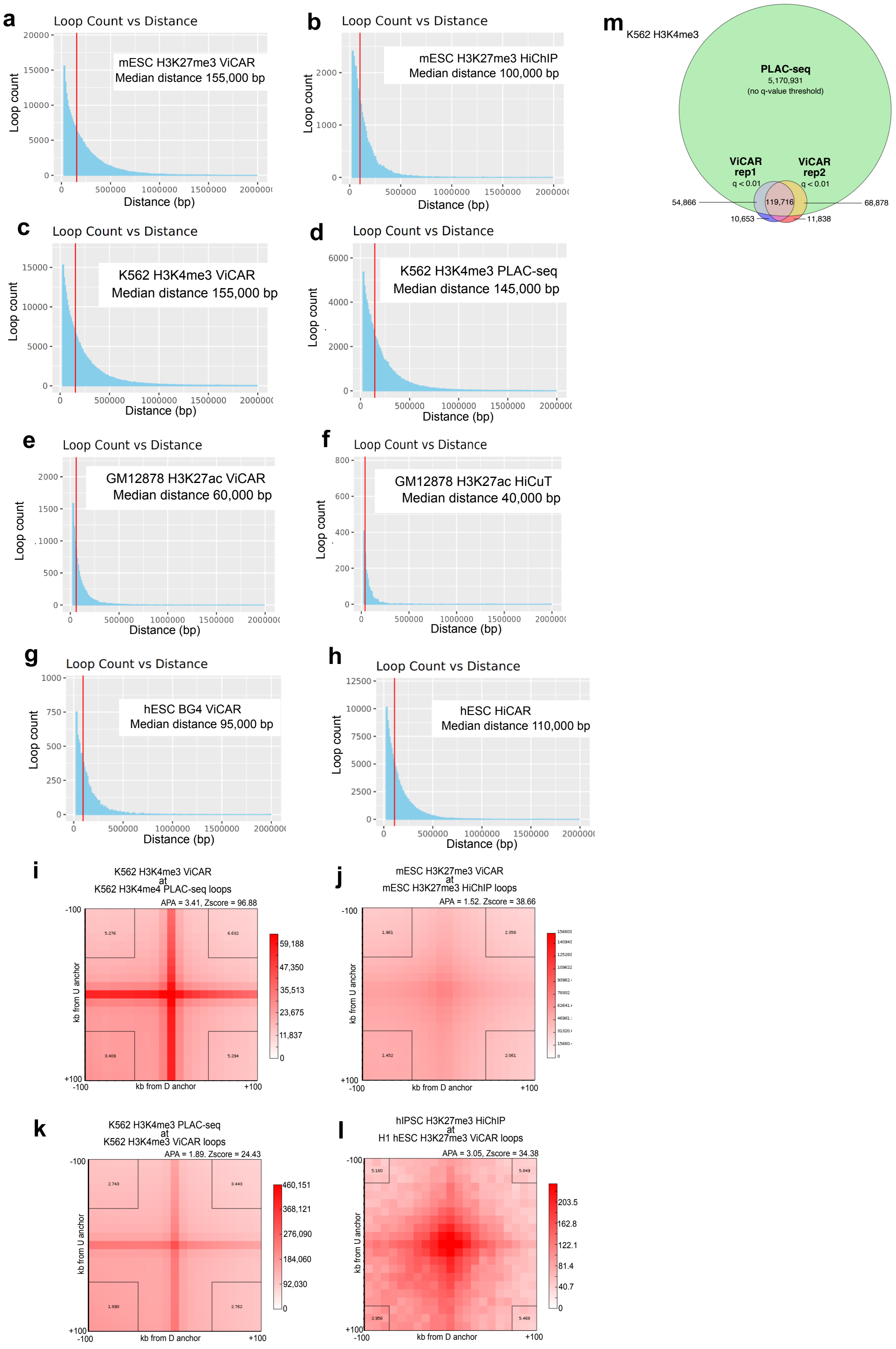

Fig S4. a-h Size distributions of loops in ViCAR, HiChIP, PLAC-seq, HiCuT and HiCAR data. **i-l** APA plots for PLAC-seq [24] loops using ViCAR data (**i**) and vice versa (**k**), and APA plots for HiChIP [23] loops using ViCAR data (**j**) and vice versa

(**l**). **m** Related to Additional File 1: Fig. S3c and d. Comparison of H3K4me3 ViCAR and PLAC-seq in K562 cells. Significant H3K4me3 ViCAR loops (q < 0.01) and all PLAC-seq interactions without significance thresholding are shown.

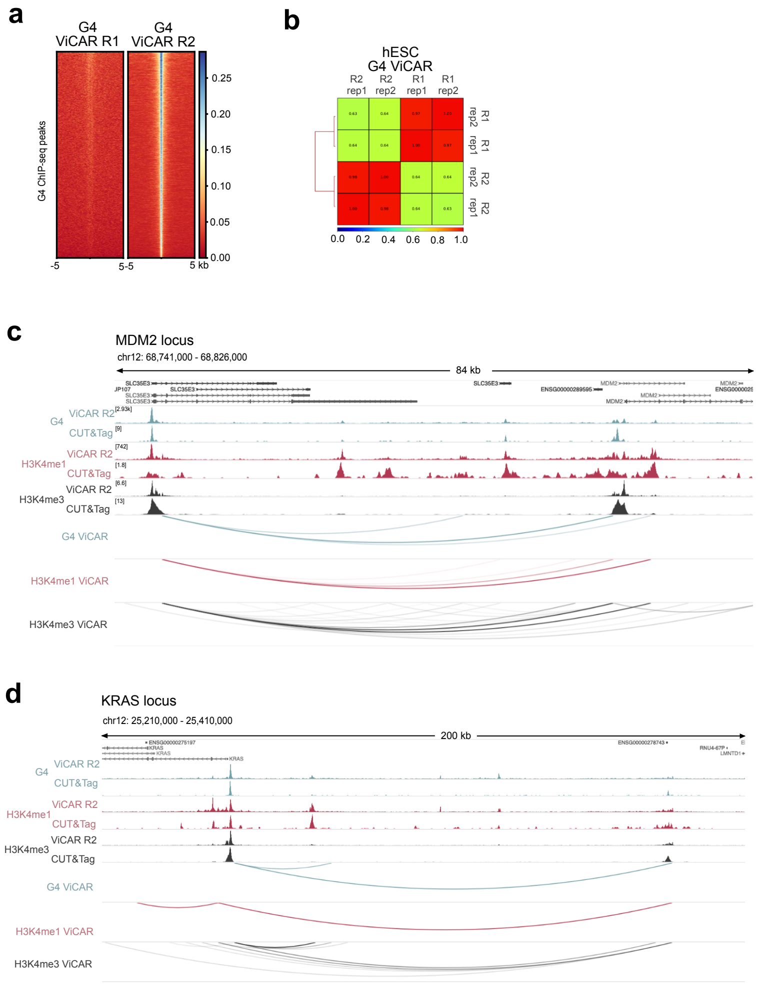

Fig S5. a ViCAR sequencing R1 and R2 plotted over G4 ChIP-seq peaks [17] in hESCs. **b** Reproducibility of ViCAR sequencing reads 1 and 2 (R1 and R2) for G4 ViCAR for two replicates (rep1 and rep2) in hESCs. Pearson correlations are shown. **c** and **d** ViCAR data from K562 cells, showing a G4-containing loops previously highlighted by Yuan et al [19] for MDM2 (**c**) and KRAS (**d**). 2D tracks show ViCAR R2 and ChIP-seq for the same mark in the same cell type, and the bottom 3 tracks show loops called by FitHiChIP ($q < 0.05$).

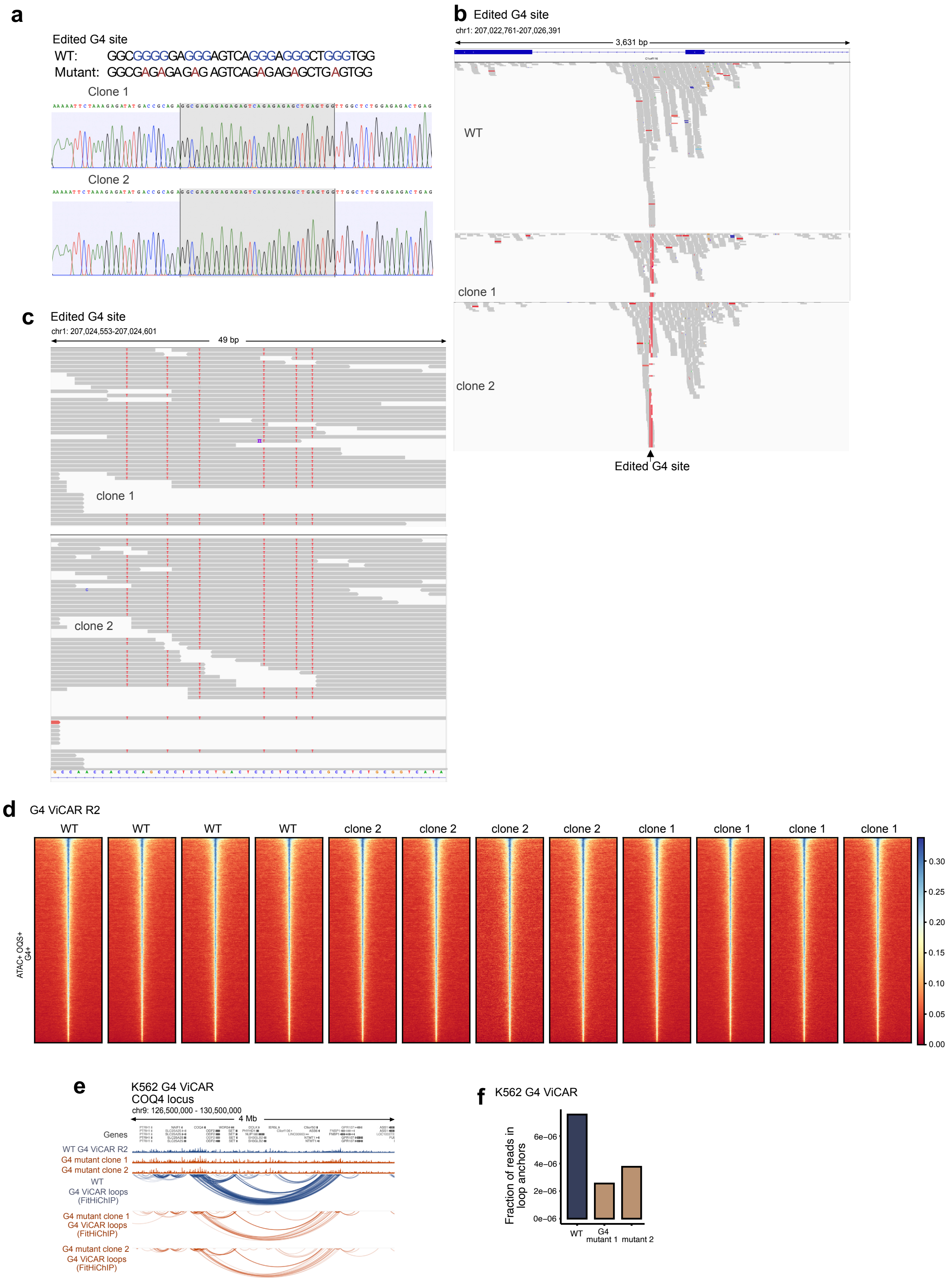

Fig S6. a Sanger sequencing confirming homozygous editing by CRISPR of a selected G4 in two independent clones (K562 cells). **b** and **c** Next Generation Sequencing of CUT&Tag libraries provides further confirmation of homozygous

editing of the targeted G4 and no off-target mutations in the surrounding ~1kb. C>T mismatches at the 6 targeted positions are visible in red. **d** G4 ViCAR R2 signal is plotted over regions that have ATAC, OQS and G4 ChIP-seq peaks genome-wide in WT and 2 G4 mutant clones. **e** Related to Fig. 2e. G4 ViCAR data from K562 cells at an unedited G4 site in WT and 2 G4 mutant clones. **f** Related to Fig. 2e. The number of reads underlying loops involving the edited site in the RCA locus (chr1:207,020,000-207,030,000) as a fraction of total number of reads per library is shown.

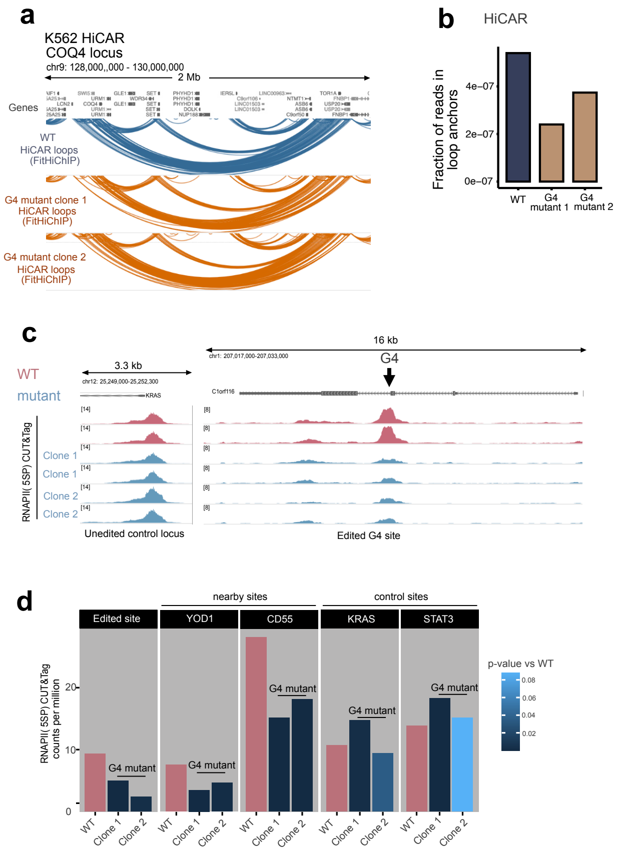

Fig S7. a Related to Fig. 2h-i. HiCAR data from K562 cells at an additional unedited site in WT and 2 G4 mutant clones. **b** Related to Fig. 2h. The number of reads underlying loops involving the edited site in the RCA locus (chr1:207020000-207030000) as a fraction of total number of reads per library is shown. **c** and **d** CUT&Tag using antibodies for initiating RNAPII (5SP), in wild type (WT) and 2 G4 mutant clones. 2 representative replicates are shown in **c**, with the *KRAS* locus shown as an unedited control locus. In **d**, RNAPII (5SP) CUT&Tag signal is quantified over the edited locus, two putative target promoters (YOD1 and CD55) and two unedited control sites (KRAS and STAT3).
