# Supplementary material for: Improved simultaneous mapping of epigenetic features and 3D chromatin structure via ViCAR
Source: Genome Biol. 2024 Sep 3;25:237. doi: 10.1186/s13059-024-03377-6 (PMC11370281; doi:10.1186/s13059-024-03377-6)
Supplement: Supplementary file 2 — Additional file 2: Table S1. ViCAR library statistics. [file 13059_2024_3377_MOESM2_ESM.pdf]

Table S1

H1 H3K27me3 VICAR	#reads	%of Total	%of Unique Valid Pairs
Total	703,195,051		
Unmapped	39,989,080	5.69	
Single Sided Mapped	99,181,333	14.10	
Mapped	564,024,638	80.21	
Duplicates	495,444,030	70.46	
Unique Valid Pairs	68,580,608	9.75	
cis	46,362,365	6.59	67.60
trans	22,218,243	3.16	32.40
Cis > 1kb	37,182,198	5.29	54.22
Cis > 2kb	35,878,721	5.10	52.32
Cis > 4kb	34,311,325	4.88	50.03
Cis > 10 kb	31,813,020	4.52	46.39
Cis > 20kb	29,708,714	4.22	43.32
Cis > 40kb	27,443,892	3.90	40.02

	#loops (5 kb)
q < 0.05	161,819
q < 0.01	121,195
q < 0.001	89,338
q < 0.0001	70,049

mESC H3K27me3 VICAR	#reads	%of Total	%of Unique Valid Pairs
Total	623,923,183		
Unmapped	41,880,405	6.71	
Single Sided Mapped	123,165,191	19.74	
Mapped	458,877,587	73.55	
Duplicates	135,862,270	21.78	
Unique Valid Pairs	323,015,317	51.77	
cis	249,810,494	40.04	77.34
trans	73,204,823	11.73	22.66
Cis > 1kb	198,593,570	31.83	61.48
Cis > 2kb	192,670,500	30.88	59.65
Cis > 4kb	185,139,207	29.67	57.32
Cis > 10 kb	172,795,385	27.69	53.49
Cis > 20kb	161,980,027	25.96	50.15
Cis > 40kb	150,275,493	24.09	46.52

	#loops (5 kb)
q < 0.05	284,174
q < 0.01	179,381

hiPSC H3K27me3 HiChIP [23]	#reads	%of Total	%of Unique Valid Pairs
Total	95,760,315		
Unmapped	10,319,071	10.78	
Single Sided Mapped	12,412,067	12.96	
Mapped	73,029,177	76.26	
Duplicates	61,614,782	64.34	
Unique Valid Pairs	11,414,395	11.92	
cis	10,708,368	11.18	93.81
trans	706,027	0.74	6.19
Cis > 1kb	1,928,062	2.01	16.89
Cis > 2kb	1,787,465	1.87	15.66
Cis > 4kb	1,624,770	1.70	14.23
Cis > 10 kb	1,402,364	1.46	12.29
Cis > 20kb	1,249,114	1.30	10.94
Cis > 40kb	1,105,154	1.15	9.68

	#loops (5 kb)
q < 0.05	0

mESC H3K27me3 HiChIP [23]	#reads	%of Total	%of Unique Valid Pairs
Total	644,288,212		
Unmapped	108,329,835	16.81	
Single Sided Mapped	150,539,821	23.37	
Mapped	385,418,556	59.82	
Duplicates	28,357,259	4.40	
Unique Valid Pairs	357,061,297	55.42	
cis	315,644,215	48.99	88.40
trans	41,417,082	6.43	11.60
Cis > 1kb	142,942,258	22.19	40.00
Cis > 2kb	138,686,587	21.53	38.84
Cis > 4kb	130,019,280	20.18	36.41
Cis > 10 kb	117,733,540	18.27	32.97
Cis > 20kb	108,587,143	16.85	30.41
Cis > 40kb	99,671,817	15.47	27.91

	#loops (5 kb)
q < 0.05	34,597
q < 0.01	17,872

K562 H3K4me3 VICAR (rep 1)	#reads	%of Total	%of Unique Valid Pairs
Total	233,520,737		
Unmapped	15,830,464	6.78	
Single Sided Mapped	62,083,009	26.59	
Mapped	155,607,264	66.64	
Duplicates	61,871,341	26.50	
Unique Valid Pairs	93,735,923	40.14	
cis	74,205,470	31.78	79.16
trans	19,530,453	8.36	20.84
Cis > 1kb	59,540,750	25.50	63.52
Cis > 2kb	56,366,948	24.14	60.13
Cis > 4kb	52,769,858	22.60	56.30
Cis > 10 kb	47,718,215	20.43	50.91
Cis > 20kb	43,727,429	18.73	46.65
Cis > 40kb	39,529,162	16.93	42.17

	#loops (5 kb)
q < 0.05	292,391
q < 0.01	212,520
q < 0.001	149,819
q < 0.0001	113,482

K562 H3K4me3 VICAR (rep 2)	#reads	%of Total	%of Unique Valid Pairs
Total	248,209,679		
Unmapped	11,881,358	4.79	
Single Sided Mapped	37,295,330	15.03	
Mapped	199,032,991	80.19	
Duplicates	103,456,759	41.68	
Unique Valid Pairs	95,576,232	38.51	
cis	76,004,901	30.62	79.52
trans	19,571,331	7.88	20.48
Cis > 1kb	61,807,120	24.90	64.67
Cis > 2kb	58,746,148	23.67	61.47
Cis > 4kb	55,003,564	22.16	57.55
Cis > 10 kb	49,461,830	19.93	51.75
Cis > 20kb	45,135,110	18.18	47.22
Cis > 40kb	40,623,323	16.37	42.50

	#loops (5 kb)
q < 0.05	272,367
q < 0.01	197,323
q < 0.001	138,946
q < 0.0001	104,590

K562 H3K4me3 PLAC-seq [24]	#reads	%of Total	%of Unique Valid Pairs
Total	314,344,836		
Unmapped	29,533,810	9.40	
Single Sided Mapped	73,418,479	23.36	
Mapped	211,392,547	67.25	
Duplicates	18,436,908	5.87	
Unique Valid Pairs	192,955,639	61.38	
cis	161,831,713	51.48	83.87
trans	31,123,926	9.90	16.13
Cis > 1kb	141,221,325	44.93	73.19
Cis > 2kb	137,456,903	43.73	71.24
Cis > 4kb	131,479,153	41.83	68.14
Cis > 10 kb	121,039,894	38.51	62.73
Cis > 20kb	112,552,306	35.81	58.33
Cis > 40kb	103,679,427	32.98	53.73

	#loops (5 kb)
q < 0.05	99,703
q < 0.01	65,005
q < 0.001	39,848
q < 0.0001	26,602

K562 H3K4me1 VICAR	#reads	%of Total	%of Unique Valid Pairs
Total	672,256,195		
Unmapped	42,979,027	6.39	
Single Sided Mapped	220,878,212	32.86	
Mapped	408,398,956	60.75	
Duplicates	250,148,975	37.21	
Unique Valid Pairs	158,249,981	23.54	
cis	120,326,065	17.90	76.04
trans	37,923,916	5.64	23.96
Cis > 1kb	105,456,610	15.69	66.64
Cis > 2kb	101,698,870	15.13	64.26
Cis > 4kb	97,094,115	14.44	61.35
Cis > 10 kb	89,973,703	13.38	56.86
Cis > 20kb	83,892,811	12.48	53.01
Cis > 40kb	77,184,312	11.48	48.77

	#loops (5 kb)
q < 0.05	294,872
q < 0.01	200,546
q < 0.001	130,467
q < 0.0001	92,018

GM12878 H3K27ac ViCAR	#reads	%of Total	%of Unique Valid Pairs
Total	25,348,089		
Unmapped	989,785	3.90	
Single Sided Mapped	3,473,110	13.70	
Mapped	20,885,194	82.39	
Duplicates	4,133,792	16.31	
Unique Valid Pairs	16,751,402	66.09	
cis	12,446,598	49.10	74.30
trans	4,304,804	16.98	25.70
Cis > 1kb	9,721,474	38.35	58.03
Cis > 2kb	9,412,234	37.13	56.19
Cis > 4kb	9,018,059	35.58	53.83
Cis > 10 kb	8,370,576	33.02	49.97
Cis > 20kb	7,805,326	30.79	46.60
Cis > 40kb	7,182,762	28.34	42.88

	#loops (10 kb)
q < 0.05	12,904

GM12878 H3K27ac HiCuT [6]	#reads	%of Total	%of Unique Valid Pairs
Total	26,141,109		
Unmapped	1,688,258	6.46	
Single Sided Mapped	1,981,156	7.58	
Mapped	22,471,695	85.96	
Duplicates	9,959,574	38.10	
Unique Valid Pairs	12,512,121	47.86	
cis	12,183,964	46.61	97.38
trans	32,8157	1.26	2.62
Cis > 1kb	948,505	3.63	7.58
Cis > 2kb	831,131	3.18	6.64
Cis > 4kb	708,499	2.71	5.66
Cis > 10 kb	574,819	2.20	4.59
Cis > 20kb	493,621	1.89	3.95
Cis > 40kb	417,513	1.60	3.34

	#loops (10 kb)
q < 0.05	2,639

H1 hESC BG4 VICAR	#reads	%of Total	%of Unique Valid Pairs
Total	641,399,896		
Unmapped	29,024,446	4.53	
Single Sided Mapped	91,909,153	14.33	
Mapped	520,466,297	81.15	
Duplicates	308,873,274	48.16	
Unique Valid Pairs	211,593,023	32.99	
cis	143,369,857	22.35	67.76
trans	68,223,166	10.64	32.24
Cis > 1kb	112,665,955	17.57	53.25
Cis > 2kb	108,500,130	16.92	51.28
Cis > 4kb	103,656,792	16.16	48.99
Cis > 10 kb	96,445,980	15.04	45.58
Cis > 20kb	90,586,818	14.12	42.81
Cis > 40kb	84,389,758	13.16	39.88

	#loops (5 kb)
q < 0.05	9,080
q < 0.01	4,388
q < 0.001	1,949
q < 0.0001	1,012

K562 BG4 VICAR	#reads	%of Total	%of Unique Valid Pairs
Total	1,650,383,057		
Unmapped	132,866,218	8.05	
Single Sided Mapped	565,490,474	34.26	
Mapped	952,026,365	57.69	
Duplicates	599,254,591	36.31	
Unique Valid Pairs	352,771,774	21.38	
cis	261,141,651	15.82	74.03
trans	91,630,123	5.55	25.97
Cis > 1kb	219,290,191	13.29	62.16
Cis > 2kb	211,174,748	12.80	59.86
Cis > 4kb	201,175,397	12.19	57.03
Cis > 10 kb	186,171,390	11.28	52.77
Cis > 20kb	174,053,966	10.55	49.34
Cis > 40kb	161,358,296	9.78	45.74

	#loops (5 kb)
q < 0.05	26,184
q < 0.01	4,644
q < 0.001	7,545
q < 0.0001	4,530

K562 WT BG4 VICAR	#reads	%of Total	%of Unique Valid Pairs
Total	747,958,430		
Unmapped	39,446,611	5.27	
Single Sided Mapped	121,755,108	16.28	
Mapped	586,756,711	78.45	
Duplicates	444,419,432	59.42	
Unique Valid Pairs	142,337,279	19.03	
cis	105,418,627	14.09	74.06
trans	36,918,652	4.94	25.94
Cis > 1kb	87,698,260	11.73	61.61
Cis > 2kb	84,931,097	11.36	59.67
Cis > 4kb	81,429,404	10.89	57.21
Cis > 10 kb	75,823,677	10.14	53.27
Cis > 20kb	71,055,992	9.50	49.92
Cis > 40kb	66,003,755	8.82	46.37

	#loops (5 kb)
q < 0.05	11,128
q < 0.01	5,843
q < 0.001	2,830
q < 0.0001	1,547

K562 G4 mutant clone 1 BG4 VICAR	#reads	%of Total	%of Unique Valid Pairs
Total	673,953,846		
Unmapped	40,123,654	5.95	
Single Sided Mapped	123,481,574	18.32	
Mapped	510,348,618	75.72	
Duplicates	401,312,811	59.55	
Unique Valid Pairs	109,035,807	16.18	
cis	73,226,655	10.87	67.16
trans	35,809,152	5.31	32.84
Cis > 1kb	59,606,856	8.84	54.67
Cis > 2kb	57,602,904	8.55	52.83
Cis > 4kb	55,189,093	8.19	50.62
Cis > 10 kb	51,543,020	7.65	47.27
Cis > 20kb	48,568,836	7.21	44.54
Cis > 40kb	45,463,761	6.75	41.70

	#loops (5 kb)
q < 0.05	2,782
q < 0.01	1,423
q < 0.001	624
q < 0.0001	336

K562 G4 mutant clone 2 BG4 VICAR	#reads	%of Total	%of Unique Valid Pairs
Total	594,735,990		
Unmapped	46,655,743	7.84	
Single Sided Mapped	139,268,856	23.42	
Mapped	408,811,391	68.74	
Duplicates	314,096,633	52.81	
Unique Valid Pairs	94,714,758	15.93	
cis	63,636,298	10.70	67.19
trans	31,078,460	5.23	32.81
Cis > 1kb	50,416,197	8.48	53.23
Cis > 2kb	48,665,014	8.18	51.38
Cis > 4kb	46,490,593	7.82	49.08
Cis > 10 kb	43,125,879	7.25	45.53
Cis > 20kb	40,324,369	6.78	42.57
Cis > 40kb	37340812	6.28	39.42

	#loops (5 kb)
q < 0.05	2,909
q < 0.01	1,529
q < 0.001	769
q < 0.0001	426

K562 WT HiCAR	#reads	%of Total	%of Unique Valid Pairs
Total	355,277,815		
Unmapped	17,743,143	4.99	
Single Sided Mapped	56,385,128	15.87	
Mapped	281,149,544	79.14	
Duplicates	118,797,956	33.44	
Unique Valid Pairs	162,351,588	45.70	
cis	123,926,571	34.88	76.33
trans	38,425,017	10.82	23.67
Cis > 1kb	99,823,945	28.10	61.49
Cis > 2kb	96,534,296	27.17	59.46
Cis > 4kb	92,324,161	25.99	56.87
Cis > 10 kb	85,394,352	24.04	52.60
Cis > 20kb	79,288,921	22.32	48.84
Cis > 40kb	72,565,891	20.43	44.70

	#loops (5 kb)	#loops (10 kb)
q < 0.05	113,343	290,691
q < 0.01	69,314	190,134

K562 G4 mutant clone 1 HiCAR	#reads	%of Total	%of Unique Valid Pairs
Total	353,590,492		
Unmapped	20,240,546	5.72	
Single Sided Mapped	60,673,716	17.16	
Mapped	272,676,230	77.12	
Duplicates	118,210,362	33.43	
Unique Valid Pairs	154,465,868	43.68	
cis	112,357,607	31.78	
trans	42,108,261	11.91	
Cis > 1kb	89,853,267	25.41	
Cis > 2kb	86,455,863	24.45	
Cis > 4kb	82,216,564	23.25	
Cis > 10 kb	75,525,679	21.36	
Cis > 20kb	69,877,491	19.76	
Cis > 40kb	63,773,258	18.04	

	#loops (5 kb)	#loops (10 kb)
q < 0.05	92,548	243,735
q < 0.01	55,525	156,474

K562 G4 mutant clone 2 HiCAR	#reads	%of Total	%of Unique Valid Pairs
Total	352,965,278		
Unmapped	19,148,240	5.72	
Single Sided Mapped	56,519,820	17.16	
Mapped	277,297,218	77.12	
Duplicates	111,762,290	33.43	
Unique Valid Pairs	165,534,928	43.68	
cis	125,514,490	31.78	75.82
trans	40,020,438	11.91	24.18
Cis > 1kb	101,259,188	25.41	61.17
Cis > 2kb	97,733,696	24.45	59.04
Cis > 4kb	93,152,158	23.25	56.27
Cis > 10 kb	85,634,948	21.36	51.73
Cis > 20kb	79,056,626	19.76	47.76
Cis > 40kb	71,765,271	18.04	43.35

	#loops (5 kb)	#loops (10 kb)
q < 0.05	127,034	305,183
q < 0.01	78,041	200,466

H1 HiCAR (Wei et al [5]).	#reads	%of Total	%of Unique Valid Pairs
Total	942,177,993		
Unmapped	126,157,457	18.77	
Single Sided Mapped	91,298,377	9.69	
Mapped	724,722,159	76.92	
Duplicates	210,100,082	22.30	
Unique Valid Pairs	514,622,077	54.62	
cis	295,170,036	31.33	57.36
trans	219,452,041	23.29	42.64
Cis > 1kb	216,280,558	22.96	42.03
Cis > 2kb	205,951,810	21.86	40.02
Cis > 4kb	193,993,927	20.59	37.70
Cis > 10 kb	177,518,856	18.84	34.49
Cis > 20kb	165,619,541	17.58	32.18
Cis > 40kb	153,674,414	16.31	29.86

	#loops (5 kb)
q < 0.05	144,206
q < 0.01	82,899
q < 0.001	45,104
q < 0.0001	28,009
