# Supplementary material for: Improved simultaneous mapping of epigenetic features and 3D chromatin structure via ViCAR
Source: Genome Biol. 2024 Sep 3;25:237. doi: 10.1186/s13059-024-03377-6 (PMC11370281; doi:10.1186/s13059-024-03377-6)
Supplement: Supplementary file 4 — Additional file 4: Table S3. Oligonucleotide sequences. [file 13059_2024_3377_MOESM4_ESM.pdf]

Table S3

Splint oligo
ACACGACGCTCTTCCGATCT

ViCAR i701 primer
caagcagaagacggcatacgagatTCGCCTTAGTCTCGTGGGCTCGGAGATGTGTATAAGAGACAG

ViCAR i702 primer
caagcagaagacggcatacgagatCTAGTACGGTCTCGTGGGCTCGGAGATGTGTATAAGAGACAG

ViCAR i703 primer
caagcagaagacggcatacgagatTTCTGCCTGTCTCGTGGGCTCGGAGATGTGTATAAGAGACAG

ViCAR i704 primer
caagcagaagacggcatacgagatGCTCAGGAGTCTCGTGGGCTCGGAGATGTGTATAAGAGACAG

ViCAR i705 primer
caagcagaagacggcatacgagatAGGAGTCCGTCTCGTGGGCTCGGAGATGTGTATAAGAGACAG

ViCAR i501 primer
AATGATACGGCGACCACCGAGATCTACACTATAGCCTACACTCTTTCCCTACACGACGCTCTTCCG
ATCT

ViCAR i502 primer
AATGATACGGCGACCACCGAGATCTACACATAGAGGCACACTCTTTCCCTACACGACGCTCTTCC
GATCT

ViCAR i503 primer
AATGATACGGCGACCACCGAGATCTACACCCTATCCTACACTCTTTCCCTACACGACGCTCTTCCG
ATCT

ViCAR i504 primer
AATGATACGGCGACCACCGAGATCTACACGGCTCTGAACACTCTTTCCCTACACGACGCTCTTCC
GATCT

ViCAR i505 primer
AATGATACGGCGACCACCGAGATCTACACAGGCGAAGACACTCTTTCCCTACACGACGCTCTTCC
GATCT

sgRNA 1 for enhancer G4 mutation (forward)
CACCGGAGATATGACCGCAGAGGCG

sgRNA 1 for enhancer G4 mutation (reverse)
AAACCGCCTCTGCGGTCATATCTCC

sgRNA 2 for enhancer G4 mutation (forward)
CACCGCTAAAGAGATATGACCGCAG

sgRNA 2 for enhancer G4 mutation (reverse)
AAACCTGCGGTCATATCTCTTTAGC

WT enhancer G4 for *in vitro* biophysical analyses
GGCGGGGGAGGGAGTCAGGGAGGGCTGGGTGG

Mutant enhancer G4 for *in vitro* biophysical analyses
GGCGAGAGAGAGAGTCAGAGAGAGCTGAGTGG
