# Supplementary material for: Improved simultaneous mapping of epigenetic features and 3D chromatin structure via ViCAR
Source: Genome Biol. 2024 Sep 3;25:237. doi: 10.1186/s13059-024-03377-6 (PMC11370281; doi:10.1186/s13059-024-03377-6)
Supplement: Supplementary file 5 — Additional file 5: Table S4. Number of replicates for sequencing experiments. [file 13059_2024_3377_MOESM5_ESM.pdf]

Table S4

Replicates

Experiment (ViCAR)	#replicates merged
H1 BG4 ViCAR	2
H1 H3K27me3 ViCAR	2
mESC H3K27me3 ViCAR	4
GM12878 H3K27ac ViCAR	3
K562 BG4 ViCAR	4
H3K4me1 ViCAR	3
H3K4me3 ViCAR rep 1	-
H3K4me3 ViCAR rep 2	-
K562 WT BG4 ViCAR	4
K562 G4 mutant clone 1 BG4 ViCAR	4
K562 G4 mutant clone 2 BG4 ViCAR	4

Experiment (HiCAR)	#replicates merged
K562 WT HiCAR	4
K562 G4 mutant clone 1 HiCAR	4
K562 G4 mutant clone 2 HiCAR	4

Experiment (other)	#replicates
RNA POL2 (5SP) CUT&Tag	7
RNA-seq	4
