# Supplementary material for: Improved simultaneous mapping of epigenetic features and 3D chromatin structure via ViCAR
Source: Genome Biol. 2024 Sep 3;25:237. doi: 10.1186/s13059-024-03377-6 (PMC11370281; doi:10.1186/s13059-024-03377-6)
Supplement: Supplementary file 6 — Additional file 6. Methods [57–64]. [file 13059_2024_3377_MOESM6_ESM.pdf]

Methods

Cell culture

K562 (ATCC) and GM12878 (Coriell) cells were cultured in RPMI1640 (Glutamine plus, Life Technologies) with 10% fetal bovine serum (Life Technologies) at 37 °C with 5% CO2. E14TG2a mESC cells [44] (kindly provided by Wolf Reik, Altos Cambridge Institute of Science, UK) were cultured on plates coated with gelatin (G9391, Sigma) in serum/LIF medium. Serum/LIF medium contained DMEM high glucose (D6546, Sigma) with 10% fetal bovine serum (16141079, Gibco), 2 mM GlutaMax-I (35050-038, Gibco), 1× NEAA512 (11140-035, Gibco), 0.1 mM β-mercaptoethanol (M3148, Sigma; diluted in EDTA/PBS) and 1,000 U/mL LIF (250-02, PeproTech). H1 hESCs (WiCell) were cultured on Matrigel-coated (#354277, Corning) 6 well plates, in mTESR1 media (#85850, STEMCELL Technologies) supplemented with 10 μM ROCK inhibitor Y-27632 for the first 24 h (#1254, Tocris). Media was changed after 1 or 2 days. H1 cells were passaged using ReLeSR (#05872, STEMCELL Technologies). Cell cultures were routinely checked for mycoplasma infection. Cell lines were not further authenticated. hESC work was authorised by the Steering Committee for the UK Stem Cell Bank and for Use of Stem Cells (UKRI).

ViCAR

Bead preparation

ViCAR was performed on beads [37], using 15 μL MyOneT1 beads per sample. For 4 samples, 60 μL MyOneT1 beads were washed 3x with 500 μL PBS pH6.8 and resuspended in 60 μL PBS pH 6.8 with 0.01% Tween-20. 30 μL biotin-conjugated con A solution (2.3 mg/ml, Sigma Aldrich C2272) were added to the bead suspension, and binding performed at room temperature (RT) for 30 min with rotation (400 rpm). After 30 min, supernatant was removed on a magnet, and beads were washed once with 60 μL PBS pH 6.8 with 0.01% Tween-20, and twice with 1 mL binding buffer (20 mM HEPES pH 7.5, 10 mM KCl, 1 mM $CaCl_2$ and 1 mM $MnCl_2$). After the last wash, beads were resuspended in 60 μL binding buffer.

Fixation, nuclei preparation, antibody binding and tagmentation

CUT&Tag steps were adapted from Hui et al [13]. For each sample, 200,000-500,000 cells were fixed in 1 mL 1% formaldehyde (Thermo Fisher Scientific, 28906) in PBS for 10 min at RT with rotation. Formaldehyde was quenched by addition of glycine to a final concentration of 0.2 M, and samples were incubated at RT for 10 min. Cells were spun for 4 min at 1,300 x g at 4 °C, supernatant removed, cells resuspended in 1 mL PBS, and then pelleted again by spinning for 4 min at 1,300 x g at 4 °C. To prepare nuclei, cells were resuspended in 1 mL NE1 buffer (20 mM HEPES pH 7.5, 10 mM KCl, 0.5 mM spermidine (S0266-5G), 0.1% Triton X-100, 20% glycerol, plus 1 Roche Complete Protease Inhibitor EDTA-Free tablet), and incubated on ice for 10 min. Nuclei were pelleted by spinning for 4 min at 1,300 x g at 4 °C, supernatant removed, and cells washed in 1 mL PBS. Nuclei were pelleted again by spinning for 4 min at 1,300 x g at 4 °C, supernatant removed, and nuclei

resuspended in 150 μL wash buffer (20 mM HEPES pH 7.5, 150 mM KCl, 0.5 mM spermidine, plus 1 Roche Complete Protease Inhibitor EDTA-free tablet). We added 15 μL conA-MyOneT1 beads (prepared above) to and samples incubated for 10 min at RT, with rotation at 800 rpm. Beads were then immobilized on a magnet, supernatant removed, and samples washed twice in 100 μL antibody buffer (2 mM EDTA, 1% BSA (Sigma-Aldrich A8577), 0.05% digitonin (EMD Milipore 300410), 20 mM HEPES pH 7.5, 150 mM KCl, 0.5 mM spermidine plus 1 Roche Complete Protease Inhibitor EDTA-free tablet). After the second wash, beads were resuspended in 100 μL antibody buffer, and blocked for 1 h at RT with rotation at 800 rpm. After 1 h, primary antibodies were added. For the following antibodies, 2 μL antibody were added to 100 μL sample: anti-H3K27me3 (Cell Signaling Technology 9733), H3K4me3 (Abcam ab8580), anti-H3K4me1 (Thermo Fisher Scientific 710795), anti-H3K27ac (Abcam ab4729). For BG4, 4 μL 1.3 μM antibody prepared in-house [11] were added to 100 μL sample. Primary antibody incubation was performed at 4 °C overnight, with rotation at 600 rpm. The next day, supernatant was removed on a magnet, and beads washed twice with 100 μL dig-wash buffer (0.05% digitonin, 20 mM HEPES pH 7.5, 150 mM KCl, 0.5 mM spermidine plus 1 Roche Complete Protease Inhibitor EDTA-free tablet). For BG4 samples, 4 μL anti-FLAG antibody (Cell Signaling Technology 2368) in 100 μL dig-wash buffer (wash buffer with 0.05% digitonin) were added to beads with incubation for 1 h at RT, shaking at 800 rpm. BG4 samples were then washed 3x in 100 μL dig-wash buffer. Next, for all samples, 1 μL anti-Rabbit IgG antibody in 100 μL dig-wash buffer were added (Antibodies-Online ABIN101961), and samples were rotated at 800 rpm at RT for 1 h. Samples were washed 3x in dig-wash buffer, then resuspended in 50 μL of a 1:250 dilution of 2 μM pA-Tn5 in dig-300 buffer (0.01% digitonin, 20 mM HEPES pH 7.5, 300 mM KCl, 0.5 mM spermidine plus 1 Roche Complete Protease Inhibitor EDTA-free tablet). pA-Tn5 was prepared in-house [13] and loaded with ME-rev and BfaI-truseqR1-pmeI-nextera as described in Wei et al [5]. pA-Tn5 binding was performed at RT for 1 h with shaking at 800 rpm. Samples were then washed 3x in 100 μL dig-300 buffer, resuspended in 100 μL tagmentation buffer (dig-300 buffer with 10 mM MgCl2), and tagmentation was performed for 1 h at 37 °C, shaking at 800 rpm. Nuclei were washed twice in 200 μL TAPS wash buffer (10 mM TAPS, 0.2 mM EDTA) on a magnet, then washed twice in 300 μL 0.075% BSA in PBS.

Hi-C preparation, CviQI digestion and in situ ligation

Remaining steps were adapted from Wei et al [5]. Beads were resuspended in 50 μL solution containing 32.5 μL nuclease-free water, 5 μL 10X NEBuffer r3.1 (NEB B6003S) and 12.5 μL 2% SDS and incubated at 62 °C for 10 min without rotation. Solution containing 100 μL nuclease-free water, 14 μL 10X NEBuffer r3.1, 25 μL 10% Triton X-100 was added (139 μL total), with incubation at 37 °C for 15 min, shaking at 800 rpm. Supernatant was removed on a magnet and beads resuspended in 300 μL 1.1X NEBuffer r3.1. Next, supernatant was removed on a magnet and beads resuspended in 101 μL solution containing: 90 μL 1.1X NEBuffer r3.1, 10 μL CviQI (NEB, R0639L), 1 μL splint oligo [5] (Additional File 4: Table S3). Samples

were incubated for 3 h at 25 °C, with rotation at 800 rpm. After 3 h, 374 µL solution containing 283.6 µL nuclease-free water, 40 µL 10% Triton X-100, 2.4 µL 20 mg/mL BSA (NEB B9000S), 48 µL 10X T4 ligation buffer were added, followed by 6 µL T4 DNA ligase. Samples were mixed by pipetting, and *in situ* ligation was performed overnight at 16 °C with interval shaking.

Reverse crosslinking and DNA purification
The next day, beads were captured on a magnet and supernatant was removed. Beads were resuspended in 100 µL solution containing 1 mg/mL Proteinase K, 0.5% SDS and 10 mM Tris-HCl buffer pH 8.0. Samples were vortexed and incubated for 1 h at 55 ºC, then for 2 h at 68 ºC, shaking at 800 rpm. DNA was purified using DNA Clean & Concentrator-5 (Zymo D4013): sample and beads were added to 500 µL binding buffer, transferred to a spin column, washed twice following manufacturer's instructions, and DNA eluted in 20 µL elution buffer.

Library preparation
Fill-in, NlaIII digestion, circularization, and PmeI digestion were performed as described in Wei et al [5]. To fill-in: solution containing 20.5 µL nuclease-free water, 5 µL 10x rCutSmart buffer (B6004S), 4 µL 10 mM dNTP, 1.5 µL T4 DNA polymerase (NEB M0203L) were added to the DNA, and samples were incubated at RT for 30 min, followed by 20 min at 75 °C. To digest DNA, 1 µL NlaIII was added and samples were incubated at 37 °C for 1 h. DNA was purified using 0.9X AMPure XP beads, eluting DNA in 80 µL 10 mM Tris-HCl (pH 8.0). For circularization, DNA was diluted to 2 ng/µL in 469 µL nuclease-free water, then 25 µL 10x T4 ligation buffer and 6 µL T4 DNA ligase (NEB M0202L) added. Samples were incubated at RT for 2 h. After ligation, DNA was purified using DNA Clean & Concentrator-5 (Zymo D4013), by combining 500 µL sample with 1 mL binding buffer, transferring 750 µL of the mix to the spin column twice, spinning between each transfer, washing following manufacturer's instructions, and eluting in 19 µL. 2.21 µL 10X rCutSmart buffer and 1.69 µL PmeI (NEB R0560L) were added to the DNA, and digestion performed at 37 °C for 1 h.

PCR and ligation junction enrichment
PCR reactions contained: 21 µL DNA, 2 µL i7 primer, 2 µL i5 primer (Additional File 4: Table S3) and 25 µL NEBNext High-Fidelity 2X PCR Master Mix (M0541S). The i7 primer anneals to the Tn5 mosaic end adapter and the i5 primer to the splint oligonucleotide, resulting in amplification of ligated fragments. The following PCR programme was performed: step 1 72 °C 4 min, step 2 98 °C 30 s, step 3 98 °C 10 s, step 4 59 °C 30 s, step 5 72 °C 45 s, step 6 72 °C 5 min, repeating steps 3-5 for a total of 12 cycles. PCR reactions were cleaned up using 1.3X AMPure XP beads, and libraries size-selected using 0.4X followed by 1.4X AMPure XP beads. Libraries were sequenced in paired end mode using the NextSeq 500 (36 bp paired-end) or NextSeq 2000 (60 bp paired-end) with 3 dark cycles for the first 3 bp of Read 1.

ViCAR read mapping and processing
Illumina sequencing paired-end files were demultiplexed using demuxFQ (flags: -c -d -i -e -t 1 -r 0.01 -R -l 9). The quality of the resulting FASTQ files was assessed using FASTQC- version 0.11.8 and bases with a quality score below 20 were trimmed from both reads using cutadapt (cutadapt -q 20). Filtered reads were then aligned to the hg38 reference genome using bwa mem [38] with flags -SP5M. Mapped reads were parsed using the pairtools package [39] (https://github.com/mirnylab/pairtools). Alignments with MAPQ > 5 were kept for further analyses. Valid read pairs were obtained by filtering out single-sided/duplicated/unmapped/multi-mapped reads.

Loop calling
ViCAR loops were identified using FitHiChIP [27] with coverage bias correction, false discovery rate (FDR) < 0.05 or < 0.01 (see Additional File 4: Table S3), a lower genomic distance threshold of 20 kb and a maximum genomic distance of 2 Mb. 5 kb and 10 kb bin sizes were used (reported in Additional File 1: Table S1). Unless stated otherwise, data shown in figures represent 5 kb bin sizes. Published H1 HiCAR [5], mESC H3K27me3 HiChIP [23], hiPSC H3K27me3 HiChIP [23], K562 H3K2me3 PLAC-seq [24] and GM12878 H3K27ac HiCuT [6] data were re-analysed using FitHiChIP with the same settings. For all published HiCAR, HiChIP and HiCuT datasets, we re-aligned reads to the hg38 reference genome. For PLAC-seq data we used a bam file available on the 4DN data portal (4DNEXRI3VAH3). ChIP-seq data were used as a reference for peak calling for ViCAR, HiChIP, PLAC-seq and HiCuT data: only pairs involving at least one ChIP-seq peak were considered (peak-to-all).

APA plots
Aggregate peak analysis (APA) was performed using Juicer Tools using significant loops with 5 kb resolution. APA score P2LL (the ratio of the central pixel to the mean of the pixels in the lower left corner) was used to summarize the APA plots.

ViCAR replicates
2-4 replicates were performed for ViCAR experiments, and data merged for loop calling as detailed in Additional file 5: Table S4. Exact sample sizes $n = 2$ (G4 ViCAR and H3K27me3 ViCAR; Additional File 1: Fig. 1b-d and Fig. 2a-b, and H3K4me3 ViCAR in Additional File 1: Fig. S3), $n = 3$ (H3K27ac ViCAR in Fig. 1i-j, and H3K4me1 ViCAR in Additional File 1: Fig. S3), $n = 4$ (G4 ViCAR in Fig. 2c and d, and mESC ViCAR in Additional File 1: Fig. S1d).

Data visualization
Valid contact pairs were transformed to Juicer hic files with juicer_tools_1.22.01.jar. Juicebox [40] and WashU Epigenome Browser [41] used to visualize raw contact martrices. R2 signal was converted to bigwig format for visualization using deepTools bamCoverage [42] with parameters: --binSize 10 --normalizeUsing CPM. 2D bigwig tracks and FitHiChIP loops were visualized with WashU Epigenome Browser [41]. Enrichment of R1 and R2 signal around ChIP-seq peaks was

calculated using deepTools computeMatrix [42] on G4 ChIP signal and plotted as heatmaps using plotProfile function. Statistical significance was assessed using a two-sided t-test.

HiCAR in WT K562 cells and G4 mutant clones

HiCAR was performed as previously described [5]. We performed 4 replicates of HiCAR for WT and 2 mutant clones. Read mapping, read processing and loop calling were performed as above as for ViCAR. For loop calling, ATAC-seq data was used for peak-to-all analyses with FitHiChIP.

Genome Editing

GAGATATGACCGCAGAGGCG sgRNA was cloned in PX458 (Addgene #48138) as described [43]. For transfection, 500,000 K562 cells were seeded in a 6-well plate and the following day transfected with 2 µg sgRNA 4 µL 10 µM 200 bp Ultramer (IDT) knock-in template with homologous flanks, using Lipofectamine LTX (Invitrogen, A12621) as per manufacturer instructions.

Circular Dichroism (CD)

WT (GGCGGGGGAGGGAGTCAGGGAGGGCTGGGTGG) and mutant (GGCGAGAGAGAGAGTCAGAGAGAGCTGAGTGG) oligos (Merck) were dissolved in buffer containing 10 mM Tris HCl pH 7.4 with either 100 mM KCl or 100 mM LiCl. CD spectra were measured with an Applied Photophysics Chirascan circular dichroism spectropolarimeter and 1 mm path length quartz cuvettes. Measurements were performed at 25 °C with a measurement range of 220 – 320 nm, and time- per-point of 0.5 s at 1 nm intervals and 1 nm bandwidth. A smoothed average of three scans was used, with baseline corrected using a buffer-only scan.

RNA-seq

RNA from 1×10^6 cells was extracted with RNeasy Plus Mini Kit (Qiagen 74136) following manufacturer's instructions. 500 ng RNA was used for library preparation with NEBNext Ultra II Directional RNA Library Prep Kit for Illumina (E7760S), following manufacturer's instructions. Polyadenylated transcripts were enriched using the NEBNext Poly(A) mRNA Magnetic Isolation Module (NEB E7490) as per the manufacturer's protocol. Libraries were sequenced in paired-end mode on a NextSeq 2000 (60 bp paired-end). Paired-end files were demultiplexed using demuxFQ (flags: -c -d -i -e -t 1 -r 0.01 -R -l 9). FASTQC version 0.11.8 was used to assess the read quality and bases with a quality score below 20 were trimmed from both reads using cutadapt (cutadapt -q 20). Genomic annotations (gtf file) were downloaded from Gencode project portal (https://ftp.ebi.ac.uk/pub/databases/gencode/Gencode_human/release_42). Filtered paired-end reads were then aligned to the hg38 reference genome using bwa mem with default parameters. Sorted bam files were generated with SAMtools (version 1.15.1). Deduplication of the resulting mapped reads was performed with Picard MarkDuplicates (v2.18.7) (http://broadinstitute.github.io/picard/). Read quantification

was done using the featureCounts function from the Subread package. Differential gene expression analysis was performed with the DESeq2 package in R (version 1.38.0) 92 on the raw read counts. Genes with an average of fewer than 10 reads per sample were omitted from downstream analysis. Benjamini & Hochberg correction was performed with significance thresholds |log2FoldChange| > 0.5 and adjusted p-value (Padj) < 0.05. Sample size for RNA-seq: *n* = 3 (G4 mutant clone 1) *n* = 4 (WT and G4 mutant clone 2).

CUT&Tag

CUT&Tag was performed as previously described [13]. For each sample, 300,000 nuclei were lightly fixed in 1 mL 0.1% formaldehyde for 2 min, followed by formaldehyde quenching by the addition of 60 µL 1.25M glycine. Cells were spun for 4 min at 1,300 x g at 4 °C, supernatant removed, cells resuspended in 1 mL PBS, and then pelleted again by spinning for 4 min at 1,300 x g at 4 °C. To prepare nuclei, cells were resuspended in 1 mL NE1 buffer (20 mM HEPES pH 7.5, 10 mM KCl, 0.5 mM spermidine (S0266-5G), 0.1% Triton X-100, 20% glycerol, plus 1 Roche Complete Protease Inhibitor EDTA-Free tablet), and incubated on ice for 10 min. Nuclei were pelleted by spinning for 4 min at 1,300 x g at 4 °C, supernatant removed, and cells washed in 1 mL PBS. Nuclei were pelleted again by spinning for 4 min at 1,300 x g at 4 °C, supernatant removed, and nuclei resuspended in 150 µL wash buffer (20 mM HEPES pH 7.5, 150 mM KCl, 0.5 mM spermidine, plus 1 Roche Complete Protease Inhibitor EDTA-free tablet). We added 10 µL conA-MyOneT1 beads (prepared above) to samples and incubated for 10 min at RT, with rotation at 800 rpm. Beads were then immobilized on a magnet, supernatant removed, and samples washed twice in 100 µL antibody buffer (2 mM EDTA, 1% BSA (Sigma-Aldrich A8577), 0.05% digitonin (EMD Milipore 300410), 20 mM HEPES pH 7.5, 150 mM KCl, 0.5 mM spermidine plus 1 Roche Complete Protease Inhibitor EDTA-free tablet). After the second wash, beads were resuspended in 100 µL antibody buffer, and blocked for 1 h, at RT with rotation at 800 rpm. After 1 h, 1:50 anti-Ser5 phosphorylated Rpb1 antibody (Cell Signaling Technology 13523) was added, with incubation performed at 4 °C overnight with rotation at 600 rpm. The next day, supernatant was removed on a magnet, and beads washed twice with 100 µL dig-wash buffer (0.05% digitonin, 20 mM HEPES pH 7.5, 150 mM KCl, 0.5 mM spermidine plus 1 Roche Complete Protease Inhibitor EDTA-free tablet). We added 1 µL anti-Rabbit IgG antibody in 100 µL dig-wash buffer (Antibodies-Online ABIN101961), and samples were rotated at 800 rpm at RT for 1 h. Samples were washed 3x in dig-wash buffer, resuspended in 50 µL of a 1:250 dilution of adapter-loaded pA-Tn5 in dig-300 buffer (0.01% digitonin, 20 mM HEPES pH 7.5, 300 mM KCl, 0.5 mM spermidine plus 1 Roche Complete Protease Inhibitor EDTA-free tablet). Samples were then washed 3x in 100 µL dig-300 buffer, resuspended in 100 µL tagmentation buffer (dig-300 buffer with 10 mM MgCl2), and tagmentation was performed for 1 h at 37 °C, shaking at 800 rpm. Nuclei were washed twice in 200 µL TAPS wash buffer (10 mM TAPS, 0.2 mM EDTA) on a magnet, then incubated for 1

h at 55 °C, shaking at 800 rpm, in 100 µL buffer containing 0.5mg/mL Proteinase K, 0.5% SDS, and 10 mM Tris-HCl pH 8.0.

A total of 9 PCR cycles were performed and libraries were sequenced in paired-end mode on a NextSeq 2000 (60 bp paired-end). Libraries were demultiplexed using demuxFQ (flags: -c -d -i -e -t 1 -r 0.01 -R -l 9). FASTQC- version 0.11.8 was used to assess the read quality and bases with a quality score below 20 were trimmed from both reads using cutadapt (cutadapt -q 20). Reads were aligned to the hg38 reference genome using bwa mem with default parameters. Sorted bam files with duplicates removed were generated with SAMtools (version 1.15.1) and converted to bigwig using deepTools bamCoverage (parameters: --normalizeUsing CPM, --binSize 10, 5 or 3 with 12 nt smoothing function). Statistical comparisons between WT and mutants were performed by two-sided t-test, using cpm signal summed over 1 kb regions. Sample size for CUT&Tag: $n = 7$.
