# Supplementary material for: Improved simultaneous mapping of epigenetic features and 3D chromatin structure via ViCAR
Source: Genome Biol. 2024 Sep 3;25:237. doi: 10.1186/s13059-024-03377-6 (PMC11370281; doi:10.1186/s13059-024-03377-6)
Supplement: Supplementary file 7 — Additional file 7. Review history. [file 13059_2024_3377_MOESM7_ESM.docx]

Review History

**First round of review**

**Reviewer 1**

**Were you able to assess all statistics in the manuscript, including the appropriateness of statistical tests used?**

No, I do not feel adequately qualified to assess the statistics.

**Comments to author:**

In this manuscript, the authors describe an approach for mapping 3D chromatin interactions associated with epigenetic features such as histone marks and G-quadruplexes (G4). Their method, ViCAR, aims to increase signal-to-noise (and therefore decrease read requirements) by coupling CUT&TAG to ligation of proximal fragments. The authors demonstrate their approach using antibodies for histone marks as well as G4. One greatly interesting innovation of this method is that, due to the sequential nature of CUT&TAG followed by ligation, the read belonging to the histone or G4 anchored site can be distinguished from the other genomic location involved in the loop. That said, the presented evidence demonstrating that this method is advantageous to similar existing approaches is not entirely compelling, and should be strengthened and/or clarified at various points, as we provide suggestions for below.

Major concerns:

Lines 62-65: The statement on line 64 "HiCuT... omits enrichment of ligated fragments" is confusing compared to line 62 where "the enrichment of ligated fragments observed in HiCuT" is discussed. Does HiCuT enrich ligated fragments or not, does ViCaR perform enrichment, and how exactly is this done? It was difficult to understand from the methods section when and how enrichment was performed, as it closely resembles a typical CUT&TAG protocol. Clarifying this in both text and methods would be helpful.

Lines 88-90: "ViCAR identified 11,433 significant loops, whereas no significant loops were detectable with HiChIP". This is a very confusing result given that the HiChIP paper identified approximately 40,000 H3K27me3 loops. Related to this, in the "loop calling" subsection of Results, HiCAR and PLAC-seq data reanalysis with FitHiChIP is described, but no information is given for the HiChIP dataset. Also, it is not clear whether the authors re-aligned reads from other published datasets or used existing valid-pair information. For example, one possible explanation for "0 detectable loops" is if FitHiChIP was run at 5-kb resolution on processed data for which 5-kb resolution is not available. Please double-check loop calling for accuracy and update the methods with more thorough information.

Throughout the manuscript, the number of loops called is used as the primary measure for demonstrating increased sensitivity of ViCAR. It is important to supplement this number, which can fluctuate based on many technical factors, with other analyses such as APA (across loops called for ViCAR as well as loops called by other methods) in order to more clearly reinforce that 1) the loops detected in ViCAR are bona fide; and 2) interactions detected by other methods are also enriched in ViCAR data. In particular, APA may facilitate compelling comparisons to HiCUT even at their low depth (i.e. APA can be performed for HiCUT samples over loops called by ViCAR).

Line 121: What is the explanation for the relatively low number of loops called in G4 ViCAR data - 9,080 vs 161,819 H3K27me3 loops called - especially given that G4 ViCAR had a much larger number of valid pairs? Is this due to the specificity of BG4 antibody, or are there other aspects of G4 formation that simply result in higher background? If G4s are widely distributed resulting in diffuse signal, are there loop calling-independent methods that could be used to further analyze G4 related interactions at promoters?

Line 128-129: "Our data... identifies new G4-3D loops." Could the authors expand on this, as it potentially represents one of the more novel findings? What loops are identified by G4 ViCAR that do not simply validate correlation from previous datasets, i.e. loops that were already marked by G4 ChIP? Does the G4 R2 data identify previously unknown G4 sites?

Figure 2c-d: It is unclear what the purpose of showing H3K4me3 and H3K4me1 data is for this figure, where the emphasis is on the consequences of G4 sequence editing. Conversely, it would be very valuable to include the G4 ViCAR R2 for mutant clones, which would visually complement the results in 2e showing loss of the G4 peak over the edited site.

The G4 editing experiment is a compelling demonstration that removing a G4 sequence affects local looping. However, according to Table S1, both mutant clones seem to identify far fewer loops than the WT - only about 25% the number called in WT - when only a handful of loops should in theory be affected. Even in Figure 2d, at the control COQ4 locus, this decrease in loops is visually apparent. Is there a potential explanation for what could be causing this, especially given that number of loops called is used throughout the manuscript as the main metric for measuring sensitivity? Does combining reads from mutant clones, or otherwise normalizing the number of valid pairs used for loop calling, help mitigate the disparity? If this issue is unsolvable, is there a greater reduction in loops at RCA from WT to mutants compared to the remainder of the genome?

Minor issues:

Figure 1a: Consider, in the legend, discussing in further detail the methodology resulting in specificity of R1 and R2, as this is one of the most intriguing aspects of ViCAR and deserves additional emphasis.

Figure 1b-c: These panels are quite confusing when following the text. The first section discusses H3K27me3 results only as a comparison to existing methods, before proceeding to discussion of ViCAR using BG4 antibody to identify G4s interactions. However, panels b and c not only combine results from both experiments, but present G4 on the left, which is challenging to follow. This figure would likely be improved as separate figures with an order following the flow of the text.

Figure S2d-f: These are not referenced in the manuscript text. Additionally, when discussion is added, please comment on whether the loop distances identified are expected compared to typical called loop sizes from HiCAR/HiChIP-like methods.

Figure S3e: It is unclear what the purpose is of examining PLAC-seq "loops" with no q-value thresholding. Does this represent something other than pure background of the PLAC-seq dataset?

Line 109: Is "cis interactions >1 kb" the best definition of reads useful to Hi-C? The supplementary table seems to highlight >20 kb as the threshold (and this percentage is still very good for the ViCAR samples).

Figure 2e: This is referenced in the text before Fig 2c-d; consider reordering.

Line 145: The reference to Fig S5d does not seem to support a "reduction in G4 structure". Is the intent of S5d to show that the reduction is specific to the locus while global G4 is unchanged?

Figure S5d: The figure legend appears incorrect, as there is no (G4-) section in the heatmap.

**Reviewer 2**

**Were you able to assess all statistics in the manuscript, including the appropriateness of statistical tests used?**

Yes, and I have assessed the statistics in my report.

**Comments to author:**

In this manuscript, the authors introduced a new method for mapping epigenomic feature-linked long-range DNA interactions. By comparing with previously published data, the authors demonstrated that ViCAR enables the discovery of much more H3K27me3- and H3K4me1-linked DNA loops than PLAC-Seq and HiChIP-Seq. They also showed that ViCAR yields enhanced signal-to-noise ratio and reduced sequencing depth requirements compared to previously reported methods. Moreover, the authors conducted endogenous G4 disruption experiments to validate the method and shed light on the role of G4 structures in DNA long-range interactions. Overall, the method should be of interest to the genome biology community for understanding the regulatory mechanisms of long-range DNA interactions.  In addition, the experiments were carefully executed, the data were nicely presented, and the manuscript was well written. I recommend the acceptance of the manuscript for publication in *Genome Biology* after some revisions and clarifications:

1. The ViCAR results for H3K27me3 displayed much better correlation between R1 and R2 than those BG4 (Figure S1a). Likewise, the ViCAR data for H3K27me3 showed a significantly higher signal-to-noise ratio compared to those for BG4. Despite a higher number of unique valid pairs in BG4 ViCAR relative to H3K27me3 (211 M vs. 68 M), fewer significant loops were identified (9 k vs. 161 k). Can the authors provide some rationale for these observations?

2. In the HiCAR paper, the authors performed signal enrichment analysis, which revealed a lack of ATAC signal enrichment in R1. A similar analysis should be conducted for BG4 ViCAR to confirm that R2 represents predominantly the G4 anchor, whereas R1 serves as the interacting anchor.

3. The disruption of G4 at selected loci resulting in the loss of G4 ViCAR signal does not necessarily substantiate the loss of long-range DNA interactions. The loss of G4 ViCAR loops could be attributed to the mere depletion of G4 structure, rendering it inaccessible to BG4 antibody during immunoprecipitation, while the genome architecture involving the edited site may remain intact. Additional experiments that do not rely on immunoprecipitation (e.g., HiCAR or HiC) should be conducted to exclude this possibility. This potential drawback may limit the applicability of the method, and should be discussed.

4.	Following the mutation of selected G4 loci and subsequent sequencing at similar depths (WT ~748M and Mut1 ~674M), G4 mut1 yielded much fewer significant loops (11,128 vs. 2,782). What explains the alteration in overall loop structures upon disruption of a specific G4 locus?

5.	While the main text portion of the manuscript was well written, some careful proof-reading for the Materials and Methods section is needed.  For instance, a number of sentences began with numbers, and no empty space was inserted between numbers and their units.

# 6. In Figure 1, very similar colors were used to denote secondary and tertiary antibodies, the same is true with T5 adaptor and overhang sequences, rendering it very difficult to visualize.  The authors may want to use colors with more pronounced differences.

**Authors Response**

**Point-by-point responses to the reviewers’ comments:**

# Reviewer 1

In this manuscript, the authors describe an approach for mapping 3D chromatin interactions associated with epigenetic features such as histone marks and G-quadruplexes (G4). Their method, ViCAR, aims to increase signal-to-noise (and therefore decrease read requirements) by coupling CUT&TAG to ligation of proximal fragments. The authors demonstrate their approach using antibodies for histone marks as well as G4. One greatly interesting innovation of this method is that, due to the sequential nature of CUT&TAG followed by ligation, the read belonging to the histone or G4 anchored site can be distinguished from the other genomic location involved in the loop. That said, the presented evidence demonstrating that this method is advantageous to similar existing approaches is not entirely compelling, and should be strengthened and/or clarified at various points, as we provide suggestions for below.

Major concerns:

1. Lines 62-65: The statement on line 64 "HiCuT... omits enrichment of ligated fragments" is confusing compared to line 62 where "the enrichment of ligated fragments observed in HiCuT" is discussed. Does HiCuT enrich ligated fragments or not, does ViCaR perform enrichment, and how exactly is this done? It was difficult to understand from the methods section when and how enrichment was performed, as it closely resembles a typical CUT&TAG protocol. Clarifying this in both text and methods would be helpful.

*Reply: HiCuT does not enrich ligated fragments, whereas ViCAR does. In ViCAR, the enrichment is achieved by PCR. One PCR primer used to generate a sequencing library anneals to the mosaic end region of the Tn5 adapter and the other anneals to a splint oligonucleotide which is ligated to genomic DNA. Therefore PCR products enrich and represent only the ligated fragments. We added the following to the text and methods:*

*Text [lines 61-64] We aimed to improve (1) the signal-to-noise seen in ChIP-based technologies by deploying CUT&Tag [6,14,22], and (2) HiCuT by enriching ligated fragments, which HiCuT does not do. HiCuT is the only CUT&Tag-based Hi-C method that we know of but only has a low proportion of useful reads (cis interactions >20 kb; Table S1) [6].*

*Text [lines 68-70] Enrichment of ligation junctions is performed via PCR using one primer that anneals to the mosaic end region of the Tn5 adapter, and another that anneals to a splint oligonucleotide which is ligated to genomic DNA [5] (Fig. 1a).*

*Methods [lines 310-312] The i7 primer anneals to the Tn5 mosaic end adapter and the i5 primer to the splint oligonucleotide, resulting in amplification of ligated fragments.*

2a. Lines 88-90: "ViCAR identified 11,433 significant loops, whereas no significant loops were detectable with HiChIP". This is a very confusing result given that the HiChIP paper identified approximately 40,000 H3K27me3 loops.

*Reply: The HiChIP paper (Kraft et al.) identified ~40,000 H3K27me3 loops in mouse embryonic stem cells (mESCs) whereas our data was for human embryonic stem cells (hESCs), so the two datasets cannot be directly compared. In the same paper, Kraft et al. also performed HiChIP in human induced pluripotent stem cells (hiPSCs), for which no loops were reported. We re-analyzed their hiPSC data to compare to ViCAR in hESCs. This analysis did not identify any significant loops with HiChIP, but detected 11,433 significant loops with ViCAR.*

2b. Related to this, in the "loop calling" subsection of Results, HiCAR and PLAC-seq data reanalysis with FitHiChIP is described, but no information is given for the HiChIP dataset. Also, it is not clear whether the authors re-aligned reads from other published datasets or used existing valid-pair information. For example, one possible explanation for "0 detectable loops" is if FitHiChIP was run at 5-kb resolution on processed data for which 5-kb resolution is not available. Please double-check loop calling for accuracy and update the methods with more thorough information.

*Reply: We re-aligned the reads from the published human dataset, and ran FitHiChIP at 5kb resolution. We down-sampled our human ViCAR data to the same number of reads and ran FitHiChIP with the same parameters. We have added this information to the loop calling section of the methods:*

*[Lines 334-339]* *Published H1 HiCAR [5], mESC H3K27me3 HiChIP [23], hiPSC H3K27me3 HiChIP [23], K562 H3K2me3 PLAC-seq [24] and GM12878 H3K27ac HiCuT [6] data were reanalysed using FitHiChIP with the same settings. For all published HiCAR, HiChIP and HiCuT datasets, we re-aligned reads to the hg38 reference genome. For published PLAC-seq data we used a bam file available on the 4DN data portal (4DNEXRI3VAH3).*

*To further address the reviewer’s point that comparison of ViCAR and existing methods in our first submission were not entirely compelling, we have now performed additional work on H3K27me3 ViCAR in mESCs. This enabled us to directly compare ViCAR to the published mESC loops referred to by the reviewer. We sequenced mESC H3K27me3 libraries to 623 million reads and using FitHiChIP detected 284,174 loops at 5 kb resolution (q < 0.05). We realigned the published mESC HiChIP from Kraft et al. (644 million reads). Using FitHiChIP with the same settings we detected 34,597 loops in the published data at 5 kb resolution (q < 0.05). Therefore, ViCAR provides increased sensitivity for detecting H3K27me3 loops in mESCs as well as hiPSCs.*

*We have added the following text:*

*[Lines 88-90] In mESCs, ViCAR identified 284,174 significant loops, whereas published HiChIP [23] only identified 34,597 at a similar sequencing depth (Fig. 1e-f and Table S1).*

*We have added the new mESC data and analyses to Table S1:*

3. Throughout the manuscript, the number of loops called is used as the primary measure for demonstrating increased sensitivity of ViCAR. It is important to supplement this number, which can fluctuate based on many technical factors, with other analyses such as APA (across loops called for ViCAR as well as loops called by other methods) in order to more clearly reinforce that 1) the loops detected in ViCAR are bona fide; and 2) interactions detected by other methods are also enriched in ViCAR data. In particular, APA may facilitate compelling comparisons to HiCuT even at their low depth (i.e. APA can be performed for HiCuT samples over loops called by ViCAR).

*Reply: We have performed APA for HiCuT vs ViCAR, PLAC-seq vs ViCAR and HiChIP vs ViCAR, comparisons.*

*To allow us to perform the suggested HiCuT vs ViCAR comparison, we have now performed an additional experiment by using H3K27ac ViCAR in GM12878 cells, since published HiCuT data was not available for the cell types in our study. To compare HiCuT to ViCAR, we re-aligned published GM12878 H3K27ac HiCuT data and processed it in the same way as ViCAR data. ViCAR data were down-sampled to a similar number of reads as HiCuT (~25 million reads for ViCAR, ~26 million reads for HiCuT). Using FitHiChIP we identified 12,904 loops with ViCAR and 2,639 loops with HiCuT (10kb resolution, q < 0.05). This demonstrates that ViCAR indeed provides increased sensitivity in terms of loop calling compared to HiCuT, and enabled us to perform aggregate peak analysis (APA). APA plots show that HiCuT data is enriched for ViCAR loops (Fig. 1j, APA = 2.66), and that ViCAR enriches HiCuT loops (Fig. 1i, APA = 3.05).*

*Using APA, we also compared ViCAR to PLAC-seq and HiChIP data. ViCAR data was enriched for PLAC-seq and HiChIP loops (Fig. S4i-j), suggesting that interactions detected by other methods are also enriched in ViCAR data. Additionally, ViCAR loops were enriched in PLAC-seq and HiChIP data (Fig. S4k-l), suggesting that loops identified by ViCAR are bona fide.*

*We have added the following text and figures to the manuscript:*

*[Line 111-118] To compare ViCAR to HiCuT directly, we performed ViCAR in GM12878 cells using the H3K27ac antibody, and down-sampled it to a similar number of reads for published HiCuT H3K27ac data in the same cell type [6] (Table S1). Whereas in HiCuT data 1.89% of total reads were useful for Hi-C (cis interactions > 20 kb), 30.79% of total reads met the same criteria in ViCAR data (Table S1). Using FitHiChIP we identified 12,904 loops with ViCAR, compared to 2,639 loops with HiCuT (10 kb resolution, q < 0.05; Table S1). Therefore ViCAR provides increased sensitivity and represents a significant advance compared to HiCuT.*

*[Line 122-130] We also used Aggregate peak analysis (APA) [29] to compare enrichment of sites identified by ViCAR and other methods. APA plots showed that HiCuT [6], PLAC-seq [24] and HiChIP [23] loops were enriched in ViCAR data (Fig. 1i and Fig. S4i-j), suggesting that interactions detected by other methods are also identified by ViCAR. ViCAR loops were also enriched in HiCuT, PLAC-seq and HiChIP data (Fig. 1j and Fig. S4k-l) indicating that ViCAR loops are bona fide. Additionally, loops that were unique to H3K4me3 ViCAR were identified in H3K4me3 PLAC-seq upon removal of a q-value threshold (Fig. S4m), supporting this conclusion [please see also point 10 below].*

***Figure 1****.* ***i*** *APA plot for GM12878 H3K27ac HiCuT [6] loops using GM12878 H3K27ac ViCAR data.* ***j*** *APA plot for GM12878 H3K27ac ViCAR loops using GM12878 H3K27ac HiCuT data [6].*

***Figure S4****.* ***i-l*** *APA plots for PLAC-seq [24] loops using ViCAR data (****i****) and vice versa (****k****), and APA plots for HiChIP [23] loops using ViCAR data (****j****) and vice versa (****l****).*

4. Line 121: What is the explanation for the relatively low number of loops called in G4 ViCAR data - 9,080 vs 161,819 H3K27me3 loops called - especially given that G4 ViCAR had a much larger number of valid pairs? Is this due to the specificity of BG4 antibody, or are there other aspects of G4 formation that simply result in higher background? If G4s are widely distributed resulting in diffuse signal, are there loop calling-independent methods that could be used to further analyze G4 related interactions at promoters?

*Reply: In our view a major contributing factor is that G4s peaks are less abundant in the genome than H3K27me3 peaks. In hESCs H3K27me3 ChIP-seq (ENCODE) peaks are ~5x broader (mean = 1298 bp, median* *= 519 bp) than BG4 peaks (mean = 197bp, median = 104 bp [17]). Additionally, there are ~1.46X more H3K27me3 peaks (26,245) compared to G4 ChIP-seq peaks (17,950) in hESCs [17].*

*Therefore ~7-fold more of the human pluripotent genome is marked by H3K27me3 than G4s, and it is therefore not unexpected that we obtain fewer loops with G4s.*

*Our goal was to identify high-confidence G4-3D loops. Therefore sites outside of G4 ChIP-seq peaks were filtered out by FitHiChIP (peak-to-all setting), and contribute to valid pairs but do not contribute to the number of loops.*

5a. Line 128-129: "Our data... identifies new G4-3D loops." Could the authors expand on this, as it potentially represents one of the more novel findings? What loops are identified by G4 ViCAR that do not simply validate correlation from previous datasets, i.e. loops that were already marked by G4 ChIP?

*Reply: By “new G4-3D loops” we intended to mean loops that have not been shown to simultaneously contain both a G4 structure and a loop. Rather, they have been independently correlated with G4s by overlapping separate Hi-C and ChIP-seq datasets. We have changed the wording in the text to clarify this.*

*[Lines 147-148] Overall, our data confirms the existence of G4s at previously predicted sites.*

*[Lines 187-188] ViCAR confirms previously predicted loops and robustly captures G4-3D interactions genome-wide.*

*As described in the methods, we used FitHiChIP’s “peak-to-all” setting to call loops. This ensures that only loops with at least one end overlapping a G4 ChIP-seq peak are considered by the loop caller. Therefore, G4 ViCAR should capture known loops (from Hi-C data) at sites that were independently shown to contain G4s (by G4 ChIP-seq). We have removed the claim that ViCAR “identifies new G43D loops”, which was misleading.*

5b. Does the G4 R2 data identify previously unknown G4 sites?

*Reply: G4 ViCAR R2 data does not capture previously unknown G4 sites. As shown below, the enrichment of G4+ and G4- sites (as defined by G4 ChIP-seq peaks) is similar in G4 ViCAR R2 and previous G4 ChIP-seq data [17].*

*Accessible chromatin regions (ATAC+) that have been experimentally shown to be capable of forming a G4 in vitro (OQS) are shown. G4 ViCAR R2 and G4 ChIP-seq are plotted over sites with G4 ChIP-seq peaks (G4+) and those without G4*

*ChIP-seq peaks (G4-). G4 ViCAR R2 signal and G4 ChIP-seq signal are comparably distributed across these two types of site.*

6. Figure 2c-d: It is unclear what the purpose of showing H3K4me3 and H3K4me1 data is for this figure, where the emphasis is on the consequences of G4 sequence editing. Conversely, it would be very valuable to include the G4 ViCAR R2 for mutant clones, which would visually complement the results in 2e showing loss of the G4 peak over the edited site.

*Reply: As suggested, we have removed the unnecessary H3K4me3 and H3K4me1 data, and added the G4 ViCAR R2 tracks for the mutant clones (Fig. 2e-f) as shown below.*

***Figure 2. e-f*** *G4 ViCAR data from K562 cells at the edited G4 site in WT and 2 G4 mutant clones. In* ***e*** *the edited G4 site is highlighted by orange shading, and genes affected by the G4 mutation (****j****) are highlighted by blue shading. 2D tracks in* ***e*** *show G4 ViCAR R2, and the bottom 3 tracks show loops called by FitHiChIP at 10 kb resolution (q < 0.01).* ***f*** *G4 ViCAR R2 signal in WT and 2 G4 mutant clones at the edited site. The edited G4 motif is shown by a black bar.*

7. The G4 editing experiment is a compelling demonstration that removing a G4 sequence affects local looping. However, according to Table S1, both mutant clones seem to identify far fewer loops than the WT - only about 25% the number called in WT - when only a handful of loops should in theory be affected. Even in Figure 2d, at the control COQ4 locus, this decrease in loops is visually apparent. Is there a potential explanation for what could be causing this, especially given that number of loops called is used throughout the manuscript as the main metric for measuring sensitivity? Does combining reads from mutant clones, or otherwise normalizing the number of valid pairs used for loop calling, help mitigate the disparity? If this issue is unsolvable, is there a greater reduction in loops at RCA from WT to mutants compared to the remainder of the genome?

*Reply: We observed this in two independent mutant clones. RNA-seq also shows that both clones have a similar profile of differentially expressed genes relative to WT (2993 genes in clone 1, 2039 genes in clone 2), which is consistent with the observed changes in G4-3D genome structure. We provide a list of all differentially expressed genes in Table S4.*

*One possible explanation is that loss of expression of one or several of the genes at this locus results in global changes in G4-3D genome structure. For example knockout/knockdown of YOD1 has been previously shown to disrupt cell proliferation and cell cycle (PMID: 37667382 and 30345304). Loss of expression of YOD1 and other nearby genes in the mutant clones may have complicated secondary consequences and may in part explain this observation.*

*As suggested, we explored whether the RCA locus is more affected by the mutation than the remainder of the genome. Fig. S6f shows the fraction of reads that map to loops where one end involves the edited site. This fraction is reduced in the mutant clones, suggesting that the effect of the mutation at the RCA locus is indeed greater than the genome-wide effect.*

*We added the following to the text and figures:*

*[Lines 163-167] Indeed, in edited cells we observed a reduction in G4 structure (Fig. 2e-g) and G4associated loops (Fig. 2e) at this locus in two independent clones. Global G4 levels were unchanged (Fig. S6d), and more loop loss was seen at the RCA locus compared to the remainder of the genome in the mutants (Fig. S6e-f).*

***Figure S6****.* ***f*** *Related to Fig. 2e. The number of reads underlying loops involving the edited site in the RCA locus (chr1:207,020,000-207,030,000) as a fraction of total number of reads per library is shown.*

Minor issues:

8. Figure 1a: Consider, in the legend, discussing in further detail the methodology resulting in specificity of R1 and R2, as this is one of the most intriguing aspects of ViCAR and deserves additional emphasis.

*Reply: We have added the following text to the legend:*

*[Lines 614-617] To amplify ligated fragments, an i7 primer that anneals to the Tn5 mosaic end adapter and an i5 primer that anneals to a splint oligonucleotide ligated to genomic DNA are used for PCR. The i7 ends of PCR products are sequenced as R2, and the i5 ends are sequenced as R1.*

Figure 1b-c: These panels are quite confusing when following the text. The first section discusses

H3K27me3 results only as a comparison to existing methods, before proceeding to discussion of ViCAR using BG4 antibody to identify G4s interactions. However, panels b and c not only combine results from both experiments, but present G4 on the left, which is challenging to follow. This figure would likely be improved as separate figures with an order following the flow of the text.

*Reply:*

*Reply: We have separated the figures to make the flow easier to follow. ViCAR using BG4 antibody is now presented in Fig. 2.*

9. Figure S2d-f: These are not referenced in the manuscript text. Additionally, when discussion is added, please comment on whether the loop distances identified are expected compared to typical called loop sizes from HiCAR/HiChIP-like methods.

*Reply: We have added the following additional text and figure panels to show loop sizes from ViCAR and other methods side by side.*

*[Lines 120-122] To further benchmark our method against existing approaches, we confirmed that the size of the loops identified by ViCAR are comparable to those called with HiChIP, PLAC-seq, HiCuT and HiCAR (Fig. S4a-h).*

***Figure S4****.* ***a****-****h*** *Size distributions of loops in ViCAR, HiChIP, PLAC-seq, HiCuT and HiCAR data.*

10. Figure S3e: It is unclear what the purpose is of examining PLAC-seq "loops" with no q-value thresholding. Does this represent something other than pure background of the PLAC-seq dataset?

*Reply: The purpose of this was to explore whether loops unique to the ViCAR dataset (i.e. not detected by PLAC-seq) were likely to be bona fide. We reasoned that these interactions may be present in the PLAC-seq data, but below the detection threshold of the loop caller. This is indeed mostly the case, so supports the conclusion that loops that are unique to ViCAR are bona fide and we have described this reasoning in the manuscript. We have changed “loops” to “interactions” in the figure legend, and have added the following to the text:*

*[Lines 126-130] ViCAR loops were also enriched in HiCuT, PLAC-seq and HiChIP data (Fig. 1j and Fig. S4k-l) indicating that ViCAR loops are bona fide [please see also point 3 above]. Additionally, loops that were unique to H3K4me3 ViCAR were identified in H3K4me3 PLAC-seq upon removal of a q-value threshold (Fig. S4m), supporting this conclusion.*

11. Line 109: Is "cis interactions >1 kb" the best definition of reads useful to Hi-C? The supplementary table seems to highlight >20 kb as the threshold (and this percentage is still very good for the ViCAR samples).

*Reply: We agree with the reviewer. > 20 kb is a more useful threshold than >1 kb. We have changed this in the text.*

*[Lines 113-115] Whereas in HiCuT data 1.89% of total reads were useful for Hi-C (cis interactions > 20 kb), 30.79% of total reads met the same criteria in ViCAR data (Table S1).*

12. Figure 2e: This is referenced in the text before Fig 2c-d; consider reordering.

*Reply: We have reordered this figure.*

13. Line 145: The reference to Fig S5d does not seem to support a "reduction in G4 structure". Is the intent of S5d to show that the reduction is specific to the locus while global G4 is unchanged?

*Reply: The purpose of Fig. S5d (now Fig. S6d) is indeed to show that the reduction is specific to the RCA locus while global G4 levels are unchanged. We have clarified this in the text:*

*[Line 165] Global G4 levels were unchanged (Fig. S6d)…*

14. Figure S5d: The figure legend appears incorrect, as there is no (G4-) section in the heatmap.

*Reply: We apologize for the mistake in the legend and have corrected this.*

# Reviewer 2

In this manuscript, the authors introduced a new method for mapping epigenomic feature-linked longrange DNA interactions. By comparing with previously published data, the authors demonstrated that ViCAR enables the discovery of much more H3K27me3- and H3K4me1-linked DNA loops than PLAC-Seq and HiChIP-Seq. They also showed that ViCAR yields enhanced signal-to-noise ratio and reduced sequencing depth requirements compared to previously reported methods. Moreover, the authors conducted endogenous G4 disruption experiments to validate the method and shed light on the role of G4 structures in DNA long-range interactions. Overall, the method should be of interest to the genome biology community for understanding the regulatory mechanisms of long-range DNA interactions. In addition, the experiments were carefully executed, the data were nicely presented, and the manuscript was well written. I recommend the acceptance of the manuscript for publication in *Genome Biology* after some revisions and clarifications:

1a. The ViCAR results for H3K27me3 displayed much better correlation between R1 and R2 than those BG4 (Figure S1a).

*Reply: High correlation between Read 1 and Read 2 in H3K27me3 ViCAR (Fig. S1a) reflects enrichment of H3K27me3 in both fragment sides (Fig. 1c and d). This observation is also consistent with previously reported homotypic 3D interactions marked by H3K27me3 in human embryonic stem cells [28].*

*We did not necessarily expect G4s to show the same trend. G4 ViCAR Read 1 shows little enrichment of G4 sites (Fig. S5a), and correlation between Read 1 and Read 2 is lower in G4 ViCAR (Fig. S5b). Therefore our data suggests that 3D interactions do not tend to involve G4s on both sides.*

*Both Read 1 and Read 2 showed high correlation between replicates in both G4 ViCAR and H3K27me3 ViCAR, confirming that this data is reproducible.*

1b. Likewise, the ViCAR data for H3K27me3 showed a significantly higher signal-to-noise ratio compared to those for BG4. Despite a higher number of unique valid pairs in BG4 ViCAR relative to H3K27me3 (211 M vs. 68 M), fewer significant loops were identified (9 k vs. 161 k). Can the authors provide some rationale for these observations?

*Reply: Please see response 4 to Reviewer 1 above.*

2. In the HiCAR paper, the authors performed signal enrichment analysis, which revealed a lack of ATAC signal enrichment in R1. A similar analysis should be conducted for BG4 ViCAR to confirm that R2 represents predominantly the G4 anchor, whereas R1 serves as the interacting anchor.

*Reply: We plotted G4 ViCAR R1 and R2 over G4 ChIP-seq peaks. We found weak enrichment of G4 ChIP-seq peaks in BG4 ViCAR R1 compared to R2, confirming that R2 predominantly represents the G4 anchor. We have added the following panel to Fig. S5.*

***Figure S5.*** ***a*** *ViCAR sequencing R1 and R2 plotted over G4 ChIP-seq peaks [17] in hESCs.*

3a. The disruption of G4 at selected loci resulting in the loss of G4 ViCAR signal does not necessarily substantiate the loss of long-range DNA interactions. The loss of G4 ViCAR loops could be attributed to the mere depletion of G4 structure, rendering it inaccessible to BG4 antibody during immunoprecipitation, while the genome architecture involving the edited site may remain intact. Additional experiments that do not rely on immunoprecipitation (e.g., HiCAR or HiC) should be conducted to exclude this possibility.

*Reply: We did not intend to claim that loss of G4 ViCAR signal represents the loss of long-range DNA interactions. Rather the intention of this experiment was to serve as a control, to confirm that loss of a G4 structure leads to a loss of G4 ViCAR’s ability to detect loops.*

*The reviewer’s suggestion is a good one, nevertheless. We performed HiCAR in WT and mutant cells (4 replicates for each; replicates were merged for FitHiChIP). As shown below (Fig. 2h-i and Fig. S7a), we observed reduced HiCAR loops at the RCA locus in the mutants.*

*To quantify this more precisely, we plotted the number of reads that map to loops involving the edited site as a fraction of the total number of reads in the library. Fig. S7b below demonstrates that there are relatively fewer HiCAR interactions involving the edited site in the mutant clones. This suggests that looping involving accessible chromatin is reduced at the RCA locus following G4 mutation. As HiCAR only detects interactions involving accessible chromatin, we cannot exclude the possibility that other 3D loops involving less accessible chromatin remain in the mutant.*

*We have added the following figures and text:*

***Figure 2.*** ***h*** *and* ***i*** *HiCAR data from K562 cells at the edited G4 site (****h****) and the unedited MYC locus (****i****) in WT and 2 G4 mutant clones. In* ***i*** *the edited G4 site is highlighted by orange shading, and genes affected by the G4 mutation (****j****) are highlighted by blue shading. Loops were called using FitHiChIP at 10 kb resolution (q < 0.01).*

***Figure S7****.* ***a*** *Related to Fig. 2h-i. HiCAR data from K562 cells at an additional unedited site in WT and 2 G4 mutant clones.*

***b*** *Related to Fig. 2h. The number of reads underlying loops involving the edited site in the RCA locus (chr1:207020000207030000) as a fraction of total number of reads per library is shown.*

[Lines 171-175] *To address what happens to the 3D landscape in the absence of a G4 in the mutant, we performed HiCAR. In edited cells we observed a reduction in HiCAR loops at the RCA locus, showing that 3D contacts associated with accessible chromatin are reduced upon loss of a G4 (Fig. 2h-i and Fig. S7a-b).*

3b. This potential drawback may limit the applicability of the method, and should be discussed.

*Reply: We have added the following to the text:*

*[Lines 168-171] One limitation of ViCAR, and other immunoprecipitation-based 3D methods, is the dependency on a target feature to probe looping. In cases where the target feature is not available, it is therefore necessary to independently measure changes in the underlying 3D genome.*

4. Following the mutation of selected G4 loci and subsequent sequencing at similar depths (WT ~748M and Mut1 ~674M), G4 mut1 yielded much fewer significant loops (11,128 vs. 2,782). What explains the alteration in overall loop structures upon disruption of a specific G4 locus?

*Reply: Please see response 7 to Reviewer 1 above.*

5. While the main text portion of the manuscript was well written, some careful proof-reading for the Materials and Methods section is needed. For instance, a number of sentences began with numbers, and no empty space was inserted between numbers and their units.

*Reply: We have carefully gone through the manuscript to check and correct errors.*

6. In Figure 1, very similar colors were used to denote secondary and tertiary antibodies, the same is true with T5 adaptor and overhang sequences, rendering it very difficult to visualize. The authors may want to use colors with more pronounced differences.

*Reply: We have used colours that are easier to discriminate.*

**Second round of review**

**Reviewer 1**

All our previous concerns have been addressed.

**Reviewer 2**

The authors did a nice job in addressing the comments raised from the last review, and the revised manuscript is substantially improved. I recommend the acceptance of the manuscript for publication in Genome Biology.
